# Thermoeconomic analysis of a combined supercritical CO_2_ reheating under different configurations of Organic Rankine cycle ORC as a bottoming cycle

**DOI:** 10.1016/j.heliyon.2022.e12230

**Published:** 2022-12-14

**Authors:** Guillermo Valencia Ochoa, Jorge Duarte Forero, Jhan Piero Rojas

**Affiliations:** a Engineering Faculty, Mechanical Engineering Department, Universidad del Atlántico, Carrera 30 Número 8–49, Puerto Colombia, Barranquilla 080007, Colombia; b Engineering Faculty, Civil Engineering Department, Universidad Francisco de Paula Santander, Avenida gran Colombia, No. 12E-96, Cucuta 540003, Colombia

**Keywords:** Energetic efficiency, Organic Rankine cycle, Thermo-economic indicator, Supercritical dioxide carbon Brayton cycle, Multi-objective optimization

## Abstract

Supercritical Brayton cycles have been considered as one of the technologies that present high thermal efficiencies in a wide range of energy conversion systems. Also, these systems can even increase their efficiency by incorporating a suitable bottoming cycle. In this article, a combined supercritical Brayton cycle with an Organic Rankine cycle (ORC) was analyzed. The influence of key system parameters such as the Brayton circuit high-pressure (Phigh), the turbine-1 inlet temperature (TIT), the turbine-1 efficiency ($n_{t}$), and the evaporation pressure ($P_{evap}$) on the economic indicators such as the Levelized Cost of Energy (LCOE), the Payback Period (PBP), the Specific Investment Cost (SIC), and net work (${\overset{˙}{W}}_{net}$) was studied. Besides, the effect of these parameters on the exergo-economic indicator $r_{k}$ and the relative cost difference $r_{k}$ were studied. Finally, a thermo-economic optimization of the proposed configurations was carried out. The study revealed that the turbine-1 inlet temperature (TIT) was the variable with the most significant effect on the economic and energy indicators of the configurations analyzed. The increase in the turbine temperature up to 850 °C caused a rise of 63.8% for both configurations. Also, the results revealed that the Brayton/SORC configuration presented the best economic performance compared to the Brayton/RORC system. The thermo-economic optimization revealed that temperatures above 800 °C and pressures between 25-30 MPa increase system performance. In addition, the Brayton/SORC configuration has a comparative reduced levelized energy costs and low payback periods, which makes it more attractive.

## 1 Introduction

Currently, there is a growing concern about low energy efficiency in power generation systems. It happened because of the environmental impact caused by greenhouse gas emissions and the constant demand for energy due to the continuous development of society [1], [2]. As a result, it is necessary to reduce the gap between the existing demand and the supply by considering new forms of energy generation or increasing the efficiency of current energy systems. In this sense, the supercritical Brayton cycle has emerged as a promising technology that not only seeks to meet the current energy demands of society but also takes into account the environmental impact associated with electricity generation [3], [4], [5], [6].

Nowadays, there are different types of supercritical Brayton configurations that have arisen to increase energy efficiency, such as supercritical Brayton cycle with reheating [7], supercritical Brayton cycle with heat and power cogeneration [8], supercritical Brayton cycle with a cascade of heat recovery [9], [10], and supercritical Brayton cycle with recompression [11], [12]. Moreover, these cycles are friendly and responsible for the environment. They can get high efficiencies in a variety of applications by operating at intermediate temperature levels in solar energy systems [13], [14], [15], nuclear power [16], high-temperature fuel cells [17], waste heat sources [18], [19], and coal-fired power plants [20], [21], among others.

The working fluid operates at temperature and pressure conditions above the critical values (supercritical state). It results in a better temperature profile between the heat source and the working fluid during heat addition [22]. In these systems, the pressure ratio of the supercritical Brayton cycle (SCBC) is low, while the turbine outlet temperature is high. For this reason, a heat recovery system is usually adapted to recuperate a large part of this energy and, thus, increase the system's efficiency [23], [24], [25]. However, a significant portion of the energy cannot be recovered. Therefore, the bottoming cycle is added to recuperate the waste heat load presented in the system. One of the routes to address this issue is the incorporation of Organic Rankine Cycles [26] which is another type of technology that is being widely used for heat recovery to increase the combined efficiency of the cycle [1], [27].

Studies on combined Organic Rankine Cycle (ORC) with supercritical Brayton cycle have been carried out by Pérez-Pichel et al. [28]. The authors explored the improvement potential in terms of energy efficiency of a supercritical Brayton cycle with recompression adapted to an ORC. The researchers found that the configuration can achieve thermal efficiencies up to 43.31%. Subsequently, Chacartegui et al. [29] evaluated the use of a supercritical Brayton cycle and transcritical Brayton cycle in a solar power plant considering three cycles. The first two cycles use an independent gas turbine, and the third uses a combined supercritical Brayton cycle with ORC. The authors concluded that these systems are prominent technologies for solar plants in terms of efficiency and cost with other conventional techniques. Following the same line, there is the work done by Sanchez et al. [30], which made a study about evaluating the performance of a supercritical Brayton cycle adapted to an ORC cycle using pure working fluids. The researchers found a 7% increase in the overall system efficiency over S-CO_2_ without the background cycle (ORC). They also found that using carbon dioxide at high pressures contributed to reducing turbomachine areas, positively impacting the economic costs of the system. Besarati and Goswami [31] evaluated the performance of a supercritical Brayton cycle with recompression and a supercritical Brayton cycle with partial cooling. The authors added an ORC system to each configuration for waste heat recovery. The results revealed that the supercritical Brayton cycle coupled with an organic Rankine cycle with reheating was more efficient than the other arrangements.

Zhang et al. [32] proposed a supercritical Brayton cycle integrated with an organic Rankine cycle powered by a nuclear reactor to use the maximum energy. The authors made parametric studies of the significant variables, finding an energy efficiency of 52.12%. They also concluded that efficiency could be improved through parametric system optimization. Song et al. [33] adapted a supercritical Brayton cycle with a recuperator coupled to an ORC for heat recovery. They evaluated different recovery ratios and considered the initial temperature of the heat source and the total heat load of the bottoming cycle. The results showed that the supercritical Brayton cycle coupled with the ORC system had a higher efficiency (17.7%) than the supercritical Brayton cycle without coupling (16.4%).

On the other hand, there are works oriented to studying thermal-economic optimization of these systems, such as those carried out by Mohammadi et al. [34]. The authors performed a thermodynamic and economic analysis to assess the waste energy recovery of supercritical Brayton cycle with recuperator, supercritical Brayton cycle with recompression, and supercritical Brayton cycle with partial cooling coupled to SORC and RORC. A total of six configurations were studied using different working fluids. The results showed that implementing an ORC to waste energy recovery from the intercooling stages increases the net power by 28.5%. Besides, it was found that waste energy recovery from the cooling stage is an attractive option for improving energy and economic performance. Habibi et al. [35] performed optimization of a regenerative supercritical Brayton cycle coupled with a simple organic Rankine cycle (SORC) driven by a concentrating solar power (CSP) tower. The results showed that implementing the ORC cycle generates an increase in net system power (2.75%) and exergy efficiency (2.16%) using helium as the working fluid.

Similar work was done by Akbari et al. [36], who reported an exergo-economic analysis for a combined supercritical recompression Brayton cycle with organic Rankine cycle considering eight working fluids. The authors concluded that the efficiency of the supercritical recompression Brayton cycle with organic Rankine cycle was higher than the supercritical recompression Brayton without ORC by 11.7%. The results also revealed that the highest exergetic efficiencies and lowest unit production costs for the supercritical recompression Brayton cycle with organic Rankine cycle were obtained using isobutane as the working fluid in the ORC. The effect of fluid type in the organic Rankine cycle on the overall exergo-economic performance of a Brayton cycle with recompression was carried out by Wang and Dai [37]. The results revealed that isobutane had the best energy and exergy performance among the fluids considered. Finally, Ahn et al. [38] made a thermo-economic analysis of a supercritical Brayton-ORC of a cogeneration cycle for heat and power production. They completed parametric studies to watch the effect of vital variables on the exergetic efficiency and unit cost of production. Subsequently, they performed a multi-target optimization. It was found an exergetic efficiency of 65.3% and a unit cost of production of 0.039 USD/kWh.

Based on the above review, many works covering fluid selection, optimization, economic analysis, etc., are evident. There have also been studies of modified Brayton cycles considering different approaches [39], [40], [41]. Among the various aspects in common that have come out of these investigations has been the increase in cycle complexity (cost) versus efficiency gained. Although adding compressors or reheating one state increases the complexity of the cycle, the system is relatively simple in contrast to modern multi-state axial-flow gas or steam turbine systems. For this reason, evaluating the economic impacts of Brayton configurations with a reheat is crucial to determine their performance. Much of the work has focused on studying Brayton cycles with recompression. The supercritical Brayton cycle with recompression has incorporated partial or total cooling stages [42]. However, few studies have considered the feasibility of using simple supercritical Brayton cycles with reheat from a thermo-economical point of view. For this reason, studies aimed at increasing the overall conversion efficiency of supercritical Brayton cycles with reheat coupled to ORC cycles contribute to providing helpful information on the performance when making decisions in terms of thermo-economics.

Therefore, based on the above, the main contribution of this work is to improve the thermodynamic and economic performance of a Brayton supercritical cycle with reheating using its exhaust heat through the use of an ORC as a bottoming cycle with toluene as a working fluid. Four key system parameters were considered such as turbine-1 inlet temperature, Brayton cycle high-pressure, turbine efficiency, and evaporation pressure of the ORC circuit. Parametric studies were made to determine the effect of these variables on energetic (${\overset{˙}{W}}_{net}$) and economic (LCOE, SIC, and PBP) parameters. Moreover, the behavior of exergo-economic indicators was analyzed, such as the difference in relative cost $\left(r_{k}\right)$ and exergo-economic factor. Finally, a multi-objective optimization was conducted to maximize both the thermal and economic performance of the proposed configurations.

## 2 Methodology

### 2.1 Descriptions of Brayton/SORC and Brayton/RORC configurations

**Figure 1:**
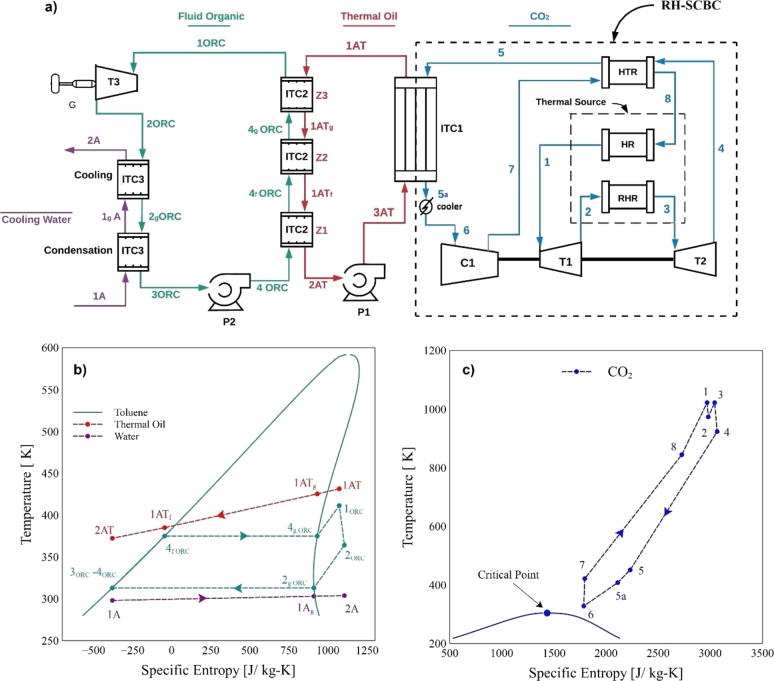
Schematic diagram: a) Brayton/SORC, b) simplified T-s diagram ORC, c) simplified T-s diagram Brayton.

Fig. 1a shows the schematic diagram of the coupling of the Brayton supercritical system with superheating to a SORC. Initially, the circuit with high-temperature and high-pressure carbon dioxide (state 1) is expanded through an intermediate pressure by an intermediate turbine (T1). The output current (state 2) is fed to the superheater (RHR), where it is reheated and then expanded to lower pressure in the second turbine (T2). The output current (state 4) is fed to the high heat exchanger (HTR), which uses its energy to heat the flow (state 7). Subsequently, the output current from the HTR (state 5) is fed to a heat exchanger (ITC1). In this step, the heat exchanger absorbs part of the heat from the carbon dioxide using a high heat transfer oil (Therminol 66). This fluid transfers the gained energy to the working fluid in the ORC evaporator. The output stream from the ITC (state 5a) passes through a cooler, which uses air to reduce the carbon dioxide temperature to 55 °C. It is then fed to the compressor, where it is compressed up to the high-pressure system (25 MPa). Subsequently, the outlet stream (state 7) enters the high-temperature recuperator (HTR), where it is preheated using the heat coming from stream 6. Finally, the outlet stream (state 8) is heated to the high-temperature of the Brayton cycle by means of the heat provided by the thermal source in the heater (HR). Fig. 1b and Fig. 1c show the thermodynamic points for the ORC and Brayton cycle, respectively

The heat absorbed by the thermal oil (state 1AT) is used as a driving force for the phase change of the organic fluid in the evaporator (ITC2). Three processes are carried out inside the ITC2 (steam generator). The first is a preheating zone (Z1), evaporation zone (Z2), and superheating zone (Z3). After passing these stages, the superheated organic fluid (state 1ORC) is fed to the ORC turbine (T3), where an expansion process occurs, generating an energy conversion in the generator (G). The turbine output current 3 (state 2ORC) passes through a condenser (ITC3) that converts the organic fluid into saturated liquid employing cooling and condensation processes using cooling water to condense all the possible mass of the organic fluid. The outlet flow from the condenser (3ORC) passes through the pump (P2), which causes a pressure increase up to the evaporating pressure of the evaporator (ITC2).

**Figure 2:**
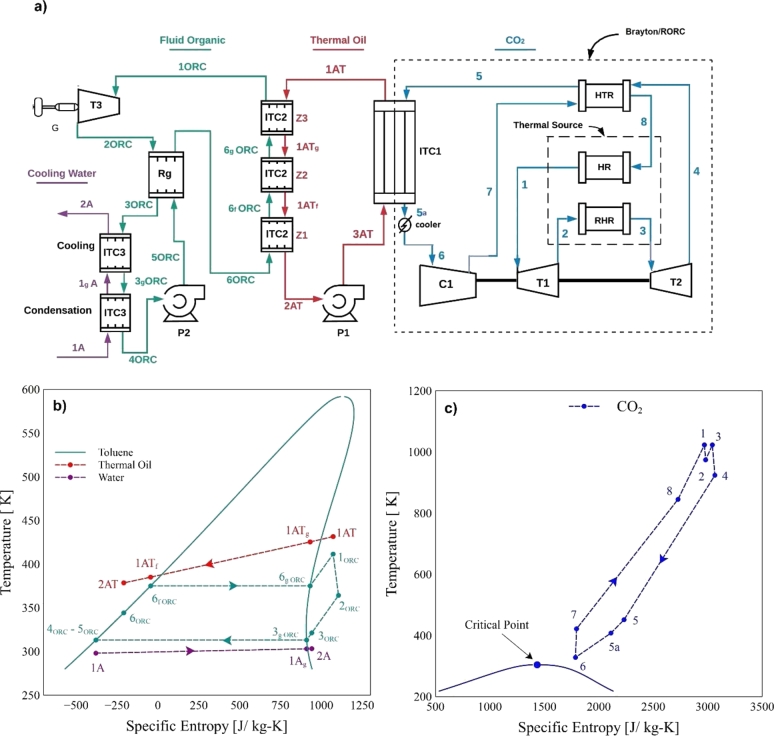
Schematic diagram: a) Brayton/RORC, b) simplified T-s diagram RORC, c) simplified T-s diagram Brayton.

This same procedure was done for the Brayton/RORC configuration, shown in Fig. 2a. Unlike the system shown in Fig. 1a, the Brayton/RORC configuration shown in Fig. 2a incorporates a heat recovery unit (Rg) to increase system efficiency. The organic fluid at the turbine output (state 2ORC) is fed to the heat recovery, which yields some energy to the output flow of pump 2 (5ORC). In this way, the ORC system recovers internal heat from the cycle by preheating the working fluid at the evaporator inlet (state 6ORC). Fig. 2b and Fig. 2c show the thermodynamic points for the ORC and Brayton cycle, respectively

### 2.2 Working fluid selection

Organic fluid selection is considered an essential step because of the wide range of fluids that have been considered and reported in the literature. Some technical, environmental, and safety restrictions have been considered in selecting the working fluid of the ORC [43]. Alkanes are hydrocarbons that have evaporation temperatures close to ambient conditions. This allows the condensation process to take place at pressures close to atmospheric pressure. In addition, their critical conditions encourage their use in high-temperature systems [44]. It is also added that alkanes are environmentally friendly, with an Ozone Depletion Potential (ODP) equal to zero and a relatively low Global Warming Potential (GWP) [45], and alkanes have been widely used in ORC applications. Table 1 shows the main environmental and thermo-physical properties of the working fluid.

**Table 1:** Environmental and thermo-physical properties of the toluene [46].

Working Fluid	Type	P_crit_ [MPa]	T_crit_ [°C]	NFPA 704		ALT [yr]	ODP	GWP
				Flammability	Health Hazard			
Toluene	Dry	4.13	319	3	2	0.007	0	2.7

### 2.3 Thermodynamic analysis

The thermodynamic analysis of the system involves the definition of the control volumes associated with the multiple components and the application of mass, energy, entropy, and exergy balances. In this way, it will also be possible to evaluate the relationships between work, heat, and associated energy and exergetic efficiency.

The simulation was performed using Matlab 2015, and the thermophysical properties of toluene and carbon dioxide at supercritical conditions were obtained by means of REPROP 9.1. The system was modeled in steady-state. Furthermore, pressure drops in piping and heat exchangers were neglected, and the expansion and compression processes were modeled adiabatically.

Consider an open stationary system using mass balance to each of the components where the input mass flow (${\overset{˙}{m}}_{in}$) will be equal to the output mass flow (${\overset{˙}{m}}_{out}$) according to equation (1).

(1) $$\sum {\overset{˙}{m}}_{in}-\sum {\overset{˙}{m}}_{out}=0$$

Similarly, the energy balance is given by equation (2).

(2) $$\sum {\overset{˙}{E}}_{in}-\sum {\overset{˙}{E}}_{out}=0$$

if heat transfer into the system is considered, the energy balance can be written according to equation (3).

(3) $${\overset{˙}{Q}}_{vc}-{\overset{˙}{W}}_{vc}+\sum {\overset{˙}{E}}_{in}h_{in}-\sum {\overset{˙}{E}}_{out}h_{out}=0$$

where $Q_{vc}$ is the heat transferred in the control volume, $W_{vc}$ is the work done within the control volume, $h_{in}$ and $h_{out}$ are the input and output enthalpies of the fluid.

On the other hand, entropy is generated due to the thermodynamic system's irreversibilities. The rate of entropy generation is obtained by equation (4), as follows [47].

(4) $${\overset{˙}{S}}_{g}+\sum \frac{{\overset{˙}{Q}}_{vc}}{T_{vc}}+\sum {\overset{˙}{m}}_{in}S_{in}-\sum {\overset{˙}{m}}_{out}S_{out}=0$$

where ${\overset{˙}{S}}_{g}$, is the entropy generated in the system, the second term is the entropy of the heat transfer in the system, and the last two correspond to the entropy due to the inlet and outlet mass flow rate. Finally, $T_{vc}$ is the temperature of the control volume.

The change of exergy can be expressed by equation (5).

(5) $${\overset{˙}{X}}_{in}-{\overset{˙}{X}}_{out}-{\overset{˙}{X}}_{D}=\Delta {\overset{˙}{X}}_{system}$$

Equation (5) can be expressed considering the heat, mass, and work is described by equation (6) [48]

(6) $$\sum (1-\frac{T_{0}}{T_{vc}})Q_{vc}-\overset{˙}{W}\sum {\overset{˙}{m}}_{in}{\overset{˙}{X}}_{in}-\sum {\overset{˙}{m}}_{out}{\overset{˙}{X}}_{out}-{\overset{˙}{X}}_{D}=0$$

where $T_{0}$, is the reference temperature, ${\overset{˙}{X}}_{D}$ is the exergy destruction rates, ${\overset{˙}{X}}_{in}$ and ${\overset{˙}{X}}_{out}$ are the input and output exergy of the fluid.

On the other hand, the energy efficiency of a system is the ratio of the system's output energy to the energy supplied to the system, expressed by equation (7).

(7) $$n_{I}=\frac{{\overset{˙}{W}}_{net}}{{\overset{˙}{Q}}_{f}}=\frac{{\overset{˙}{W}}_{net}}{\left(h_{1}-h_{8}\right){\overset{˙}{m}}_{CO2}+(h_{3}-h_{2}){\overset{˙}{m}}_{CO2}}$$

where ${\overset{˙}{Q}}_{f}$ is the heat given by thermal source to the system.

Finally, the efficiency of the second law can be obtained by equation (8)

(8) $$n_{\mathrm{II}}=\frac{{\overset{˙}{W}}_{net}}{{\overset{˙}{E}}_{In}}=\frac{{\overset{˙}{W}}_{net}}{{\overset{˙}{Q}}_{f}(1-T_{0}/T_{high})}$$

where $T_{0}$ is the reference temperature (298,15 K), and $T_{\mathrm{high}}$ is the turbine inlet temperature

### 2.4 Heat exchanger modeling

Heat exchange equipment represents about 15-20% of the system cost [5]. Therefore, calculating the heat transfer areas is crucial to evaluating the economic viability of these systems.

#### 2.4.1 Shell and tube heat exchanger

**Figure 3:**
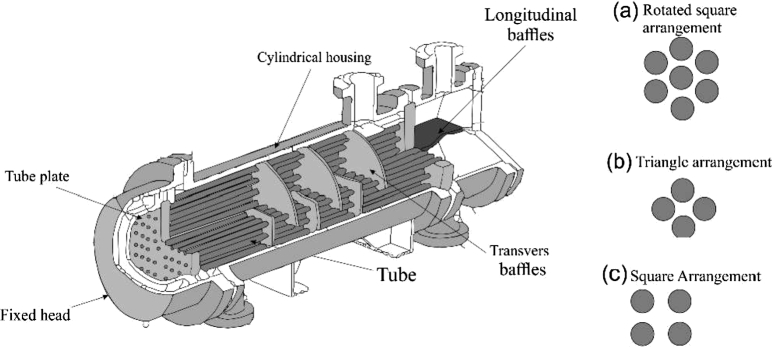
Shell and tube heat exchanger design: a) rotated square arrangement; b) triangle arrangement; c) square arrangement.

For the design of the heat exchanger (ITC1), an energy balance was applied to calculate the heat gained by the thermal oil [49]. Fig. 3 shows an illustration of the constituent parts of the equipment.

The heat absorbed by the thermal is given by equation (9)

(9) $$\overset{˙}{Q}={\overset{˙}{m}}_{CO2}C_{P,CO2}\left(T_{5}-T_{5a}\right)$$

where $\overset{˙}{Q}$ is the heat transferred, ${\overset{˙}{m}}_{CO2}$ is the mass flow rate of carbon dioxide, and $C_{P,CO2}$ is the calorific capacity of carbon dioxide.

The logarithmic mean temperature difference (LMTD) for this type of countercurrent flow exchanger can be determined by equation (10).

(10) $$LMTD=\frac{\left(T_{5}-T_{1AT}\right)-\left(T_{5a}-T_{3AT}\right)}{ln\left(\frac{T_{5}-T_{1AT}}{T_{5a}-T_{3AT}}\right)}$$

The deviation of the mean temperature variation is corrected by the factor F shown in equation (11).

(11) $$F=\left(\sqrt{\frac{R^{2}+1}{R-1}}\right)\frac{ln\left(\frac{1-P}{1-P\cdot R}\right)}{ln\left(\frac{2-P\cdot R+1-\sqrt{R^{2}+1}}{2-P\cdot R+1+\sqrt{R^{2}+1}}\right)}.$$

where *R* and *P* are the correlation factors given by equations (12) and (13).

(12) $$R=\frac{T_{5}-T_{5a}}{T_{1AT}-T_{3AT}}$$

(13) $$P=\frac{T_{1AT}-T_{3AT}}{T_{5}-T_{3AT}}$$

Also, the overall heat transfer coefficient of the exchanger is calculated by equation (14).

(14) $$\frac{1}{U_{0}}=\frac{1}{H_{ex}}+\frac{1}{H_{Eext}}+\frac{D_{ext}ln\left(\frac{D_{ext}}{D_{int}}\right)}{2K_{m}}+\left(\frac{D_{ext}}{D_{int}}\frac{1}{h_{Eint}}\right)+\left(\frac{D_{ext}}{D_{int}}\frac{1}{H_{in}}\right)$$

where $D_{ext}$, $D_{int}$ are the external and internal diameters of each pipe, $K_{f}$ is the thermal conductivity of the exhaust gases, and finally $h_{Eext}$ is the convective coefficients external side tube and $h_{ex}$ is the convective coefficients side shell.

Finally, the surface area of the heat exchanger can be determined by equation (15).

(15) $$A=\frac{\overset{˙}{Q}}{U_{0}F(LMTD)}$$

#### 2.4.2 Plate fin heat exchanger design

**Figure 4:**
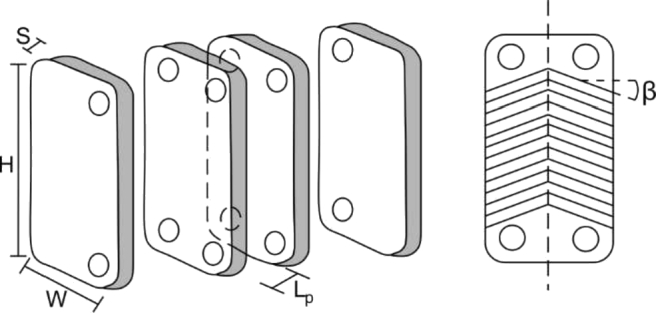
Plate fin heat exchanger.

Fig. 4 shows the geometrical parameters for the design of plate heat exchangers. The exchanger was divided into three parts called areas. Preheating area (A1), evaporation area (Z2), and reheating area (Z3).

The area of zone 1 is determined by equation (16).

(16) $$A_{Z1}=\frac{{\overset{˙}{Q}}_{Z1}}{U_{Z1\_A}LMTD_{Z1}}$$

where ${\overset{˙}{Q}}_{Z1}$ is obtained by equation (17)

(17) $${\overset{˙}{Q}}_{Z1}={\overset{˙}{m}}_{AT}\left(h_{ATf}-h_{2AT}\right)$$

where, ${\overset{˙}{m}}_{\mathrm{AT}}$ is the thermal oil flow, and $h_{2AT}$ and $h_{ATf}$ are the enthalpies. The overall heat transfer coefficient is determined by equation (18).

(18) $$U_{Z1\_C}=\frac{1}{\left(\frac{1}{h_{Z1\_AT}}+\frac{1}{h_{Z1\_ORC}}\right)}$$

where the terms $h_{Z1\_AT}$ and $h_{Z1\_ORC}$ are the convective coefficients on the thermal oil side and ORC, respectively. The LMDT is calculated by equation (19).

(19) $$LMTD_{Z1}=\frac{\left(T_{ATf}-T_{ORC}\right)-\left(T_{2AT}-T_{1ORC}\right)}{log\left(\frac{T_{ATf}-T_{ORC}}{T_{2AT}-T_{1ORC}}\right)}$$

The total heat exchanger surface area is determined by the sum of all the areas obtained.

### 2.5 Thermo economic analysis

The total production cost (*TPC*) is calculated according to equation (20) [50].

(20) TPC=TCI+O&M

where the total capital to be invested is given by equation (21).

(21) TCI=FCI+other costs

where *FCI* represents the investment of fixed assets of the system, according to equation (22). Where *DC* and *IC* are the direct costs and the indirect costs.

(22) FCI=DC+IC

The term “other costs” in equation (21) refers to the start-up costs, the initial working capital, and research and development.

**Figure 5:**
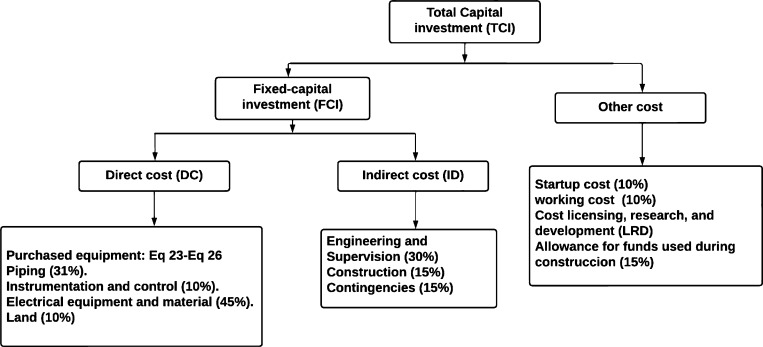
Diagram of the composition of the total capital to be invested.

Fig. 5 decomposes each of the terms for the calculation of the total capital investment (TCI). In addition, the percentages used to estimate them according to literature reports are shown. These percentages are a function of the acquisition cost of all the equipment in the system [51], [52].

Correlations based on heat exchanger area, turbine, and pump power [53], [54], [55] were used to calculate the purchased equipment cost. The cost of the turbine is given by equation (23) [53], [55]

(23) $$log_{10}(Z)=2.6259+1.4398log_{10}({\overset{˙}{W}}_{t})-0.1776{\left[log_{10}({\overset{˙}{W}}_{t})\right]}^{2}$$

for the heat exchanger [54], [55], equation (24) is used

(24) $$Z=10000+324\left(A^{0,91}\right)$$

for the pump, the cost is given by equation (25), [53], [55]

(25) $$log_{10}(Z)=3.3892+0.0536log_{10}({\overset{˙}{W}}_{p})-0.1538{\left[log_{10}({\overset{˙}{W}}_{p})\right]}^{2}$$

Finally, for the compressor [56], equation (26) is used

(26) $$Z=91562{\left(\frac{{\overset{˙}{W}}_{comp}}{445}\right)}^{0.67}$$

The levelized value of the project costs was obtained through the constant escalation leveling factor (CELF) using equation (27) [57].

(27) $$CELF=CRF\cdot \frac{k\left(1-k^{n}\right)}{1-k}$$

where *n* is the project lifetime. The parameters *k* and CRF were calculated by equation (28) and equation (29).

(28) $$k=\frac{1+r_{n}}{1+i_{eff}}$$

(29) $$CRF=\frac{i_{eff}{\left(1+i_{eff}\right)}^{n}}{{\left(1+i_{eff}\right)}^{n}-1}$$

where $i_{eff}$ is the annual interest rate of 5%, $r_{n}$ is the nominal scaling ratio of 5%, *n* is the project time of 20 years, and (*τ*) is the annual operating hours of 7446 [58].

#### 2.5.1 Cost of exergy

**Figure 6:**
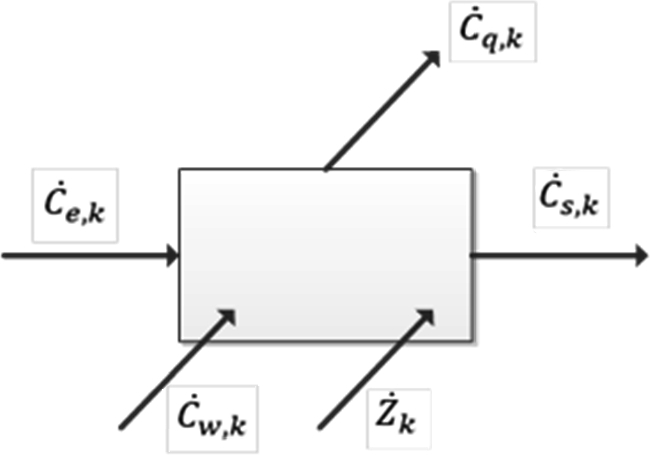
Cost balance of the economic analysis.

The first step for the thermo-economic evaluation of the cycle is the determination of the cost associated with the exergy of each of the streams of the processes. For this purpose, a cost balance was made for each component, as seen in Fig. 6, expressed according to equation (30).

(30) $$\sum_{e}{\overset{˙}{C}}_{e,k}+{\overset{˙}{C}}_{w,k}+{\overset{˙}{Z}}_{k}^{OM}+{\overset{˙}{Z}}_{k}^{CI}={\overset{˙}{C}}_{q,k}+\sum_{s}{\overset{˙}{C}}_{s,k}$$

where ${\overset{˙}{C}}_{e,k}$ and ${\overset{˙}{C}}_{s,k}$ are the costs of the inlet and outlet flows, respectively. Where ${\overset{˙}{C}}_{w,k}$ is the costs associated with power and ${\overset{˙}{C}}_{q,k}$ is the costs associated with heat transfer. ${\overset{˙}{Z}}_{k}^{OM}$ refers to level operating and maintenance costs. Finally, ${\overset{˙}{Z}}_{k}^{CI}$ refers to the leveled costs of initial investment and payment of financial responsibilities. These last two terms can be grouped into one term ${\overset{˙}{Z}}_{k}={\overset{˙}{Z}}_{k}^{OM}+{\overset{˙}{Z}}_{k}^{CI}$ for simplicity.

The above cost balance can also be presented in terms of specific costs ($c_{k})$ associated with the exergy of each of the streams (${\overset{˙}{E}}_{k}$), as well as the heat transfer and power according to equation (31).

(31) $$\sum_{e}c_{e,k}{\overset{˙}{E}}_{e,k}+c_{w,k}{\overset{˙}{W}}_{k}+{\overset{˙}{Z}}_{k}=c_{q,k}{\overset{˙}{Q}}_{k}+\sum_{s}c_{s,k}{\overset{˙}{E}}_{i,k}$$

equation (31) is applied to each component. It acquires a particular character depending on the type of energy interactions that this component has with the rest of the system and the surroundings.

The methodology proposed by Bejan is applied [48] to determine the values of ${\overset{˙}{Z}}_{k}$. The operation and maintenance costs for each equipment are estimated as a proportional value to the investment cost of the equipment according to equation (32).

(32) $${\overset{˙}{Z}}_{k}=\frac{\left({\overset{˙}{C}}_{COM}+{\overset{˙}{C}}_{CI}\right)}{PEC}PEC_{k}$$

where, ${\overset{˙}{C}}_{COM}$ is the ratio of the annual cost of operation and maintenance and the hours a cycle operates in a year. ${\overset{˙}{C}}_{CI}$ refers to the ratio of the annual cost associated with the initial investment and the number of hours that the cycle operates in the year. $PEC_{k}$ is the acquisition cost of component *k*, and *PEC* is the acquisition cost of the entire cycle. The number of hours of operation for one year is 8760 h, and the capacity factor is set to 85% [58].

To determine the cost of the power supplied to the compressor and the power delivered by the turbine, the value of the leveled cost of energy (LCE) and the net power delivered by the cycle were taken as a base according to the equation (33).

(33) $${\overset{˙}{C}}_{w,turb}=LCE({\overset{˙}{W}}_{net})$$

The LCE already includes the effect of the load factor. It is defined as the minimum value to sell the power to cover all the costs necessary for electricity generation: initial investment, operation and maintenance, and fuel costs, as well as the costs associated with the financial, legal, and tax obligations. The cost of the compressor power is calculated using equation (34).

(34) $${\overset{˙}{C}}_{w,comp}=\left(\frac{{\overset{˙}{C}}_{w,turb}}{{\overset{˙}{W}}_{turb}}\right){\overset{˙}{W}}_{comp}$$

#### 2.5.2 Cost of destroyed exergy

When the costs of the stream exergies, the costs of operation and maintenance, and initial investment are determined, it is proceeded to estimate the cost of the exergy destroyed. It was considered that part of the exergy is transferred to the products. Another part escapes to the environment through the losses, and the remaining exergy is destroyed by the entropy generated. The previous exergy balance is expressed mathematically by equation (35)

(35) $${\overset{˙}{E}}_{P,k}={\overset{˙}{E}}_{F,k}-{\overset{˙}{E}}_{loss,k}-{\overset{˙}{E}}_{D,k}$$

Bejan [48] proposes the following model to quantify the cost of exergy destroyed from the input exergy (${\overset{˙}{E}}_{F,k}$) and the output exergy (${\overset{˙}{E}}_{P,k}$) of the component, according to the equation (36).

(36) $$c_{P,k}{\overset{˙}{E}}_{P,k}=c_{F,k}{\overset{˙}{E}}_{F,k}-c_{F,k}{\overset{˙}{E}}_{loss,k}-c_{F,k}{\overset{˙}{E}}_{D,k}+{\overset{˙}{Z}}_{k}$$

From equation (36) the cost of exergy destroyed is expressed by the equation (37)

(37) $${\overset{˙}{C}}_{D,k}=c_{F,k}{\overset{˙}{E}}_{D,k}$$

where ${\overset{˙}{C}}_{D,k}$ is the cost of destroying exergy, in $/h, $c_{F,k}$ is the specific cost of exergy entering the component, and ${\overset{˙}{E}}_{D,k}$ is the rate of exergy destruction in the *k* component. The variable $c_{P,k}$ refers to the specific cost of the exergy that is removed from the system as a product. The amounts $c_{F,k}$, and $c_{P,k}$ are determined from the stream exergies and associated costs given in Table 2, using equations (38) and (39).

(38) $$c_{F,K}=\frac{{\overset{˙}{C}}_{F,K}}{{\overset{˙}{E}}_{F,K}}$$

(39) $$c_{P,K}=\frac{{\overset{˙}{C}}_{P,K}}{{\overset{˙}{E}}_{P,K}}$$

**Table 2:** Input-output cost equations, input-output exergies, and exergo-economic balance of the main components.

Components	Scheme	Cost product Cost fuel	Exergy product Exergy fuel	Cost equations
C1		${\overset{˙}{C}}_{p,C}={\overset{˙}{C}}_{7}-{\overset{˙}{C}}_{6}$ ${\overset{˙}{C}}_{f}={\overset{˙}{C}}_{w,C1}$	${\overset{˙}{E}}_{p}={\overset{˙}{E}}_{7}-{\overset{˙}{E}}_{6}$ ${\overset{˙}{E}}_{f}={\overset{˙}{W}}_{C1}$	$c_{7}{\overset{˙}{E}}_{7}+c_{W,C1}{\overset{˙}{W}}_{C1}=c_{6}{\overset{˙}{E}}_{6}+{\overset{˙}{Z}}_{C1}$
B2		${\overset{˙}{C}}_{p,P}={\overset{˙}{C}}_{4ORC}-{\overset{˙}{C}}_{3ORC}$ ${\overset{˙}{C}}_{f}={\overset{˙}{C}}_{w,P2}$	${\overset{˙}{E}}_{p}={\overset{˙}{E}}_{4ORC}-{\overset{˙}{E}}_{3ORC}$ ${\overset{˙}{E}}_{f}={\overset{˙}{W}}_{P2}$	$c_{4ORC}{\overset{˙}{E}}_{x4ORC}=c_{4ORC}{\overset{˙}{E}}_{x3ORC}+c_{WT1}{\overset{˙}{W}}_{P2}+{\overset{˙}{Z}}_{P2}$ *c*_*w*,*P*2_ = *c*_*w*,*P*2_
T1		${\overset{˙}{C}}_{p}={\overset{˙}{C}}_{w,T1}$ ${\overset{˙}{C}}_{f}={\overset{˙}{C}}_{1}-{\overset{˙}{C}}_{2}$	${\overset{˙}{E}}_{p}={\overset{˙}{W}}_{T1}$ ${\overset{˙}{E}}_{f}={\overset{˙}{E}}_{1}-{\overset{˙}{E}}_{2}$	$c_{2}{\overset{˙}{E}}_{2}+c_{WT1}{\overset{˙}{W}}_{T1}=c_{1}{\overset{˙}{E}}_{1}++{\overset{˙}{Z}}_{T1}$
T3		${\overset{˙}{C}}_{p}={\overset{˙}{C}}_{w,T3}$ ${\overset{˙}{C}}_{f}={\overset{˙}{C}}_{1ORC}-{\overset{˙}{C}}_{2ORC}$	${\overset{˙}{E}}_{p}={\overset{˙}{W}}_{T3}$ ${\overset{˙}{E}}_{f}={\overset{˙}{E}}_{1ORC}-{\overset{˙}{E}}_{2ORC}$	$c_{2ORC}{\overset{˙}{E}}_{2ORC}+c_{W,T3}{\overset{˙}{W}}_{T3}=c_{1ORC}{\overset{˙}{E}}_{1ORC}++{\overset{˙}{Z}}_{T3}$
ITC1		${\overset{˙}{C}}_{p}={\overset{˙}{C}}_{1AT}-{\overset{˙}{C}}_{3AT}$ ${\overset{˙}{C}}_{f}={\overset{˙}{C}}_{5}-{\overset{˙}{C}}_{5a}$	${\overset{˙}{E}}_{P}={\overset{˙}{E}}_{1AT}-{\overset{˙}{E}}_{EAT}$ ${\overset{˙}{E}}_{f}={\overset{˙}{E}}_{5}-{\overset{˙}{E}}_{5a}$	$c_{5}{\overset{˙}{E}}_{5}+c_{3AT}{\overset{˙}{E}}_{3AT}+{\overset{˙}{Z}}_{ITC1}=c_{5a}{\overset{˙}{E}}_{5a}+\;c_{1AT}{\overset{˙}{E}}_{1AT}$
ITC2		${\overset{˙}{C}}_{p}={\overset{˙}{C}}_{1ORC}-{\overset{˙}{C}}_{4ORC}$ ${\overset{˙}{C}}_{f}={\overset{˙}{C}}_{1AT}-{\overset{˙}{C}}_{2AT}$	${\overset{˙}{E}}_{P}={\overset{˙}{E}}_{1ORC}-{\overset{˙}{E}}_{6ORC}$ ${\overset{˙}{E}}_{f}={\overset{˙}{E}}_{2AT}-{\overset{˙}{E}}_{1AT}$	$c_{1AT}{\overset{˙}{E}}_{1AT}+c_{4ORC}{\overset{˙}{E}}_{4ORC}+{\overset{˙}{Z}}_{ITC2}$ =c2ATE˙2AT+c1ORCE˙1ORC
ITC3		${\overset{˙}{C}}_{p}={\overset{˙}{C}}_{2A}-{\overset{˙}{C}}_{1A}$ ${\overset{˙}{C}}_{f}={\overset{˙}{C}}_{2ORC}-{\overset{˙}{C}}_{3ORC}$	${\overset{˙}{E}}_{P}={\overset{˙}{E}}_{2A}-{\overset{˙}{E}}_{1A}$ ${\overset{˙}{E}}_{f}={\overset{˙}{E}}_{2ORC}-{\overset{˙}{E}}_{3ORC}$	$c_{1ORC}\left({\overset{˙}{E}}_{x3ORC}-{\overset{˙}{E}}_{x2ORC}\right)+c_{2A}{\overset{˙}{E}}_{2A}={\overset{˙}{Z}}_{ITC3}$ *c*_1*A*_ = 0
HTR		${\overset{˙}{C}}_{p}={\overset{˙}{C}}_{8}-{\overset{˙}{C}}_{7}$ ${\overset{˙}{C}}_{f}={\overset{˙}{C}}_{4}-{\overset{˙}{C}}_{5}$	${\overset{˙}{E}}_{P}={\overset{˙}{E}}_{8}-{\overset{˙}{E}}_{7}$ ${\overset{˙}{E}}_{f}={\overset{˙}{E}}_{4}-{\overset{˙}{E}}_{5}$	$c_{4}{\overset{˙}{E}}_{4}+c_{7}{\overset{˙}{E}}_{7}+{\overset{˙}{Z}}_{HTR}=c_{5}{\overset{˙}{E}}_{5}+c_{8}{\overset{˙}{E}}_{8}$

### 2.6 Thermo-economic performance indicators

The relative difference between the product-specific cost and inputs ($r_{k}$) given by equation (40) and the ex-economic factor ($f_{k}$) given by equation (41) are two factors proposed by Bejan to evaluate the performance of the system.

(40) $$r_{k}=\frac{c_{P,k}-c_{F,k}}{c_{F,k}}$$

equation (41) measures the increase in the specific cost of products concerning the particular costs of the inputs. A negative value of this indicates that the performance of the *k* component process decreases the quality of the inlet energy, reducing as well the cost of exergy production. The exergo-economic factor is calculated according to equation (41).

(41) $$f_{k}=\frac{{\overset{˙}{Z}}_{k}}{c_{F,k}{\overset{˙}{E}}_{D,k}+{\overset{˙}{Z}}_{k}}$$

### 2.7 Economic performance indicators

To evaluate the economic viability of the system given by equation (42), the leveled cost of energy (LCOE) was defined to find the minimum value of the sale of electricity produced in the system to support the investment costs.

(42) $$LCOE=\frac{\sum_{n=0}^{N}(C_{n}+O\&M_{n}+FE_{n})}{\sum_{n=0}^{N}\frac{E_{n}}{{\left(1+r\right)}^{n}}}$$

where $C_{n}$ is the cost of components, $O\&M_{n}$ are operating and maintenance expenses, $FE_{n}$ is the fuel cost, $E_{n}$ is the annual energy production, *r* is the interest rate, and *n* is the number of years of the project [59].

The Specific Investment Cost (SIC) was used to study the total investment cost and the net power of the system, expressed by equation (43) [60].

(43) $$SIC_{ORC}=\frac{C_{ORC}}{{\overset{˙}{W}}_{T}-{\overset{˙}{W}}_{P}}$$

where $C_{ORC}$ is the total cost of ORC, and ${\overset{˙}{W}}_{T}$ and ${\overset{˙}{W}}_{P}$ are the work of the turbine and pump, respectively.

Finally, the payback period (PBP) is defined according to equation (44) [61]

(44) $$PBP=\frac{C_{TDC}}{\left(1-t\right)\left(S_{annual}-C_{TPC}\right)+C_{D}}$$

Where $C_{TPC}$ is the annual production cost, $C_{TDC}$ is the total depreciable capital, $S_{annual}$ is the annual sales revenues direct costs, *D* is the annual depreciation, and *t* is the sum of federal and state income tax rate.

### 2.8 Multi-objective optimization and decision making

The Multi-objective optimization tool is a model which represents more than one objective. In this study, payback period (PBP), specific investment cost (SIC), net power (${\overset{˙}{W}}_{Net}$), and levelized cost of energy (LCOE) were selected as objective functions. Different scenarios combining the net power with the economic indicators were proposed to determine which one is the most appropriate. That is, the purpose is to find the values of the decision variables that maximize the net power at the lowest economic cost with different combinations: ${\overset{˙}{W}}_{Net}$/LCOE, ${\overset{˙}{W}}_{Net}$/SIC, and ${\overset{˙}{W}}_{Net}$/PBP. In this sense, the multiobjective optimization problem is defined by equation (19). Its constraints shown in equation (45)-(48) [46].

(45) $$\min F\left(x\right)={\left[fobj_{1}\left(X\right),fobj_{2}\left(X\right),fobj_{3}\left(X\right),\ldots fobj_{n}\left(X\right)\right]}^{T}$$

(46) x∈S,

(47) $$S=\{x\in R^{m}:M\left(x\right)=0,N\left(x\right)\geq 0\}$$

(48) $$C=\{y\in R^{n}:y=fobj(x),x\in S$$

where $fobj_{n}$ are the objective functions considered in the study, M(x), and N(x) are the constraints of the problem. In these cases, there is no single solution that globally minimizes or maximizes all the objective functions.

This tool seeks to generate a set of Pareto points for all objective functions. A Pareto optimal solution is optimal if and only if it is not dominated by any other solution. In that case, all the Pareto optimal solutions form the set of solutions called Pareto front [62].

The decision variables used in the study were: high-temperature, high-pressure, turbine efficiency, and evaporation pressure. Each of these variables is defined in a search range in the form of a vector, whose values are shown in Table 3.

**Table 3:** Lower and upper boundary of the decision variables.

Decision Variables	Lower bound	Upper bound	Unit
High-temperature	550	850	°C
High-pressure	20	30	MPa
Turbine efficiency	80	92	%
Evaporation pressure	2	18	%

For this study, optimization was carried out using the Non-Dominated Sorting Genetic Algorithm (NGSA)-II using Matlab optimization environment, which is a technique based on evolutionary theory. This technique is based on six steps: population, selection, reproduction, mutation, crossover, and migration. Table 4 shows the values used for the optimization process.

**Table 4:** Input parameters of optimization of NSGA II [63].

Description	Value
Crossover fraction	0.80
Generation size	400
Selection process	Tournament
Migration fraction	0.2
Population size	50

**Figure 7:**
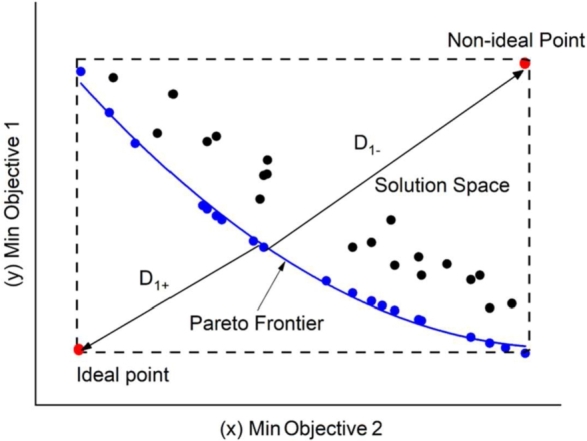
Illustration decision making approaches.

For a two-objective minimization problem, the solutions will be distributed in the solution space. Fig. 7 illustrates as an example the Pareto optimal solution (in blue) and the Pareto non-optimal solutions (in black). In this work, the TOPSIS tool (Technique of Order Preference by Similarity to and Ideal Solution) was applied. The solution with the highest value within the search space is called the non-ideal solution. The solution with the lowest value is called the ideal solution. Therefore, a solution as far away as possible from the non-ideal and as close as possible to the ideal solution must be found [62].

To calculate the Euclidean distance ($D_{i}$) between the Pareto points and the ideal and non-ideal points, equations (49) and (50) are applied.

(49) $$D_{i+}=\sqrt{\sum_{j=1}^{n}{\left(F_{ij}-F_{j}^{ideal}\right)}^{2}}$$

(50) $$D_{i-}=\sqrt{\sum_{j=1}^{n}{\left(F_{ij}-F_{j}^{non\text{-}ideal}\right)}^{2}}$$

where, $F_{j}^{ideal}$ is the positive ideal solution, and $F_{j}^{\text{non-ideal}}$ is the no-ideal solution, *i* denotes the index of each solution of the Pareto frontier, and *j* is the index of the objective functions considered in the study. The final selection is given by equation (51).

(51) $$Y_{i}=\frac{D_{i-}}{D_{i-}+D_{i+}}$$

## 3 Results and discussion

This section presents the results of the parametric study of two configurations Brayton/SORC and Brayton /RORC. The effect of the Brayton cycle turbine-1 inlet temperature, the Brayton cycle high-pressure, the turbine efficiency (turbine-1, and turbine-2 cycle Brayton), and the ORC cycle evaporation pressure on the energy indicators (${\overset{˙}{W}}_{net}$), and economic indicators (LCOE, SIC, PBP) were analyzed.

The high-temperature cycle is a parameter that significantly affects system performance allowing levels up to 850 °C [41]. The effect of system high pressure also influences thermal efficiency, equipment operating life, and levelized cost of energy [7]. Additionally, the evaporating pressure of the working fluid in the ORC is one of the most critical parameters limiting system operability. The pressure range strongly depends on the source temperature entering the evaporator [64]. Finally, the turbine efficiency of the Brayton cycle is widely studied, which influences the thermal energy generation rate. The main values used in the simulation for the configurations considered are reported in Table 5.

**Table 5:** Input parameters used for the performance of the different plant layouts.

Subsystem	Parameters	Value	Unit	References
SORC/ORC	Turbine isentropic efficiency	0.85	%	[58]
SORC/ORC	Pump isentropic efficiency	0.85	%	[58]
SORC/ORC	Cooling water temperature (T1A)	25	°C	[58]
SORC/ORC	Condensation temperature	40	°C	[58]
SORC/ORC	Evaporating pressure	78.8	kPa	[58]
SORC/ORC	Minimum temperature approximation, Ap	20	°C	[58]
SORC/ORC	Pinch Point temperature (ITC2) (ITC3)	10	°C	[58]
RORC	Effectiveness recuperator	0.85	%	[58]
SORC/ORC	Pressure ratio P1	1.5	-	[58]
SORC/ORC	Mass flow thermal oil	0.5	kg/s	[58]
Brayton	Compressor isentropic efficiency	0.89	%	[64]
Brayton	Turbine inlet temperature	750	°C	[64]
Brayton	Heat exchanger effectiveness	0.93	%	[64]
Brayton	High-Pressure Brayton Cycle	25	MPa	[64]
Brayton	Turbine isentropic efficiency	0.93	%	[64]
Brayton	Minimum pinch point temperature	5	°C	[64]
Brayton	Compressor inlet temperature	55	°C	[64]
Brayton	Mass flow CO_2_	1	kg/s	[64]

Table 6 shows data of thermodynamic properties and the exergy costs for each of the process stages using toluene as working fluid in the ORC circuit and carbon dioxide in the Brayton circuit at their base conditions reported in Table 5.

**Table 6:** Thermodynamic properties and cost of exergy stream Brayton/RORC.

State	Fluid	*T* [K]	*P* [kPa]	*h* [kJ/kg]	$\overset{˙}{E}$ [kW]	*m* [kg/s]	*c* [USD/GJ]	$\overset{˙}{C}$ [USD/h]
1	CO_2_	1023.15	25000	1285.61	710.52	1.00	86.00	192.59
2	CO_2_	971.58	17482	1222.24	644.98	1.00	86.00	165.84
3	CO_2_	1023.15	17482	1287.62	690.81	1.00	56.70	121.46
4	CO_2_	918.25	8202	1160.37	558.93	1.00	56.70	96.76
5	CO_2_	451.28	8202	613.83	259.03	1.00	56.70	53.35
5a	CO_2_	407.91	8202	562.63	243.41	1.00	56.70	53.35
6	CO_2_	328.15	8202	444.58	222.36	1.00	163.00	128.07
7	CO_2_	421.73	25000	500.84	275.83	1.00	94.90	98.52
8	CO_2_	839.95	25000	1052.90	552.50	1.00	81.30	143.11
4ORC	Toluene	313.15	7.89	-132.35	0.06	0.11	208.00	0.74
5ORC	Toluene	313.18	78.92	-132.26	0.07	0.11	299.38	1.33
6ORC	Toluene	344.04	78.92	-76.61	0.62	0.11	324.83	17.55
6fORC	Toluene	375.18	78.92	-17.04	1.71	0.11	359.86	27.36
6gORC	Toluene	375.18	78.92	349.23	9.78	0.11	274.62	51.89
1ORC	Toluene	411.28	78.92	404.04	11.20	0.11	208.00	58.93
2ORC	Toluene	363.94	7.89	337.14	2.97	0.11	208.00	26.04
3ORC	Toluene	321.30	7.89	281.50	2.20	0.11	208.00	11.61
3gORC	Toluene	313.15	7.89	271.65	2.13	0.11	208.00	9.83
1A	Water	298.15	101.33	104.92	0.00	2.07	0.00	0.00
1gA	Water	303.15	101.33	125.82	0.36	2.07	305.29	9.41
2A	Water	303.27	101.33	126.33	0.38	2.07	470.83	21.02
1AT	Thermal oil	431.28	200	305.30	21.00	0.50	211.47	50.53
1ATg	Thermal oil	425.28	200	293.12	19.24	0.50	211.47	25.59
1ATf	Thermal oil	385.18	200	214.93	9.34	0.50	211.47	6.72
2AT	Thermal oil	378.66	200	202.78	8.00	0.50	211.47	1.97
3AT	Thermal oil	378.72	300	202.91	8.02	0.50	261.82	2.46

### 3.1 Effect of the temperature, pressure, and turbine efficiency on the energy and economic indicators

**Figure 8:**
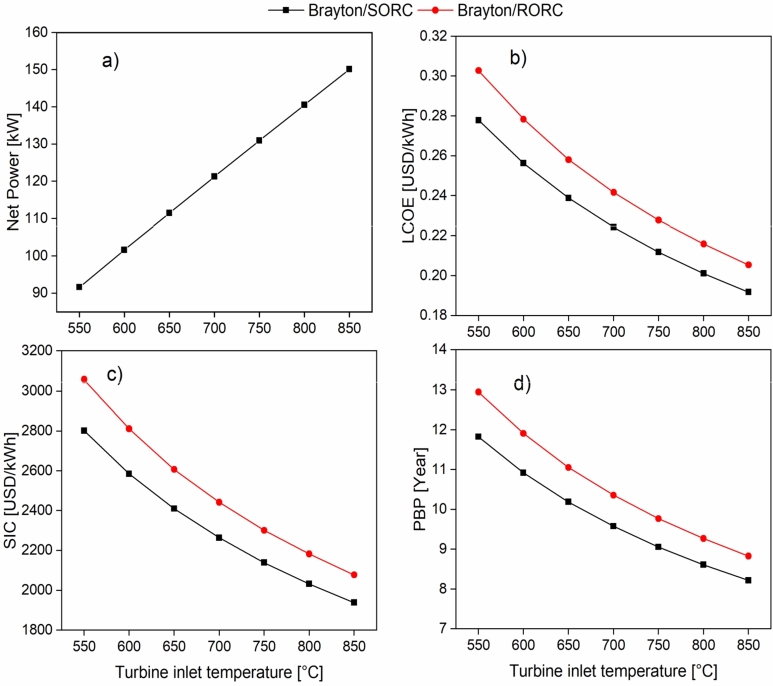
Effect of turbine-1 inlet temperature on a) Wnet; b) LCOE; c) SIC; d) PBP.

Fig. 8 shows the effect of the turbine-1 inlet temperature on the energy and economic indicators. Fig. 8a shows that the increase in turbine-1 inlet temperature from 550 °C to 800 °C generates an increase in the net combined cycle power Brayton/SORC from 91.63 kW to 150.15 kW, which is equivalent to 63.8%. It is evident that the systems have a linear behavior in the increase of power for the Brayton/SORC and Brayton/RORC configurations. It happens because the higher the temperature input of the working fluid of the Brayton cycle, the higher the amount of energy available in the transferred fuel for the production of mechanical energy.

Figs. 8b to 8d show the behavior of LCOE, SIC, and PBP as a function of the temperature. It can be seen that the increase in the inlet temperature of turbine-1 in the Brayton/SORC and Brayton/RORC configurations decreases the economic indicators. On the other hand, the higher the turbine-1 inlet temperature, the higher the net power of the cycle. It is relevant to emphasize that an increase in power generation causes an increase in the cost of acquiring the turbine. It is also observed that the Brayton/RORC system presents higher values in the economic indicators compared to the Brayton/ORC cycle. It happens because adding the heat recuperator to the ORC circuit impacts the total cost of the cycle investment. It causes a high investment recovery time, as shown in Fig. 8d. Therefore increasing the turbine-1 inlet temperature from 550 °C to 850 °C generates a decrease in LCOE of 31% and 32% for the Brayton/SORC and Brayton/RORC systems, respectively. A similar result was found for SIC, which decreased with increasing turbine-1 inlet temperature for the Brayton/SORC (30.8%) and Brayton/RORC (32%) configurations. Finally, the payback period was also strongly favored, with a reduction of 30.5% and 31.8% for Brayton/SORC and Brayton/RORC, respectively.

**Figure 9:**
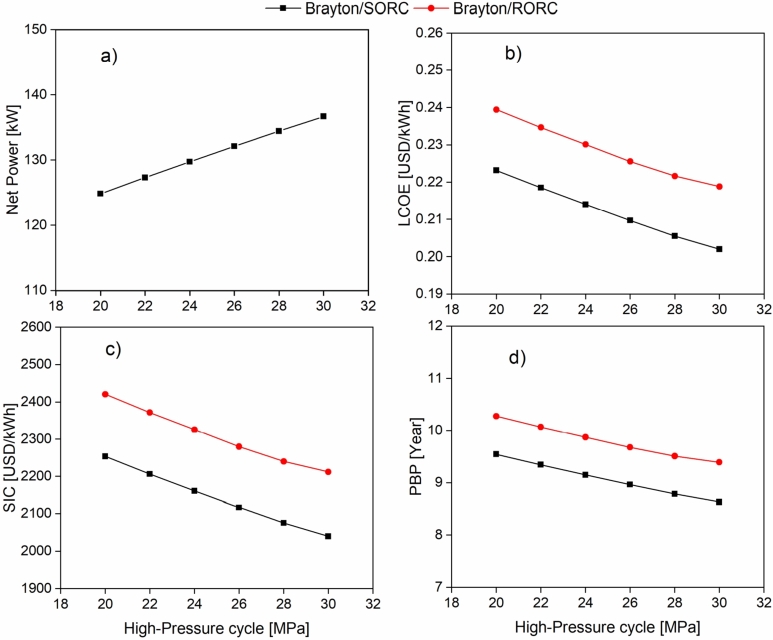
Effect of high-pressure cycle Brayton on a) Wnet; b) LCOE; c) SIC; d) PBP.

Fig. 9 shows the effect of the Brayton cycle high-pressure on Wnet, LCOE, SIC, and PBP. Fig. 9a illustrates that the increase of the high-pressure of the cycle from 20 to 30 MPa generates an increase in the net power cycle from 124 kW to 136 kW, representing an increase of 9.4% for both configurations. It is evident that the systems have a linear behavior with the increase of the high pressure of the cycle. An increase in the Brayton system's operating pressure favors a higher heat recovery rate in the heat exchanger equipment. In addition, the enthalpy jump in the turbine increases, which produces an increase in power generation. Also, Figs. 9b-9d show that economic indicators decrease when increasing the high-pressure cycle. The power generated in the system has an impact on economic indicators, causing a reduction in the LCOE (Fig. 9b) for Brayton/SORC (9.5%) and Brayton/RORC (8.6%). Similar behavior was found in the investment recovery rates (Fig. 9d), which decreased by 9.5%, and 8.6% for the Brayton/SORC and Brayton/RORC configurations, respectively. Finally, the specific investment costs (Fig. 9c) was reduced by 9.5% and 8.6% for the Brayton/SORC and Brayton/RORC configurations, respectively.

It can also be seen that the Brayton/RORC system presents the highest values in the economic indicators concerning those obtained in the Brayton/SORC configuration. It happens due to the additional cost that the Brayton/RORC configuration has for the addition of a heat recovery unit. It impacts the increase of the total investment costs of the cycle. It is appropriate to highlight that the rise of the high-pressure in the Brayton circuit promotes an enhancement in the transport properties of $CO_{2}$. It offers additional economic benefits, such as the reduction of the carbon footprint of heat transfer equipment. On the other hand, reducing the turbomachinery area implies a decrease in system costs [27], which is illustrated in Fig. 9a.

**Figure 10:**
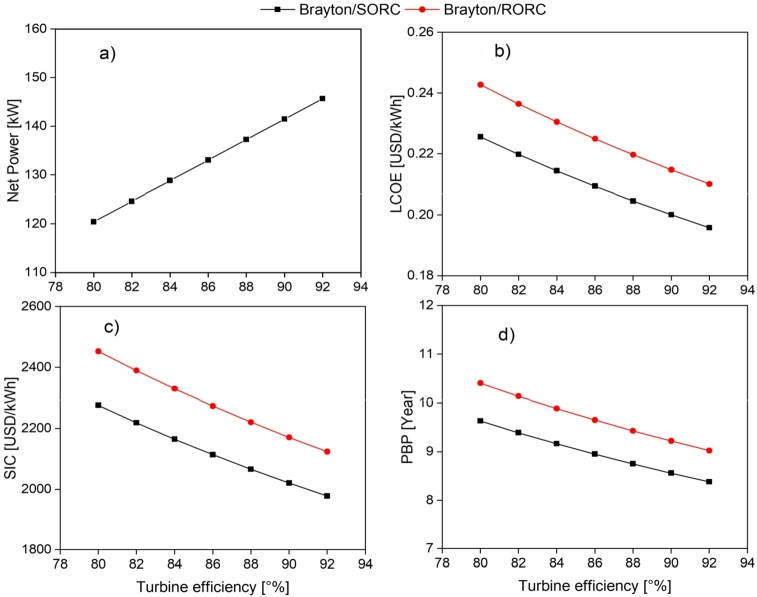
Effect of efficiency turbine on a) Wnet; b) LCOE; c) SIC; d) PBP.

Fig. 10 shows the effect of turbine-1 efficiency on the system's energy and economic indicators. Fig. 10a shows that net power tends to increase when increasing turbine efficiency. The increase is 21% for both configurations.

The linear increase of the system's net power due to the increase in the efficiency of the Brayton cycle turbines is related to the machine's better conversion of enthalpic energy to thermal energy in the expansion process. This causes that the economic parameters to decrease since power generation predominates over equipment acquisition costs. Fig. 10b shows that as efficiency increases, the levelized cost of energy decreases for the Brayton/SORC (7.1%) and Brayton/RORC (3.1%) configurations. The same tendency is found in the specific investment cost (Fig. 10c) for the Brayton/SORC (7.2%) and Brayton/RORC (3.1%) configurations. Finally, the payback period (PBP) was favored since the time in which the investment is returned was reduced, as shown in Fig. 10d. It should be noted that the Brayton/SORC configuration is the one that presents the best economic performance under the current study conditions.

**Figure 11:**
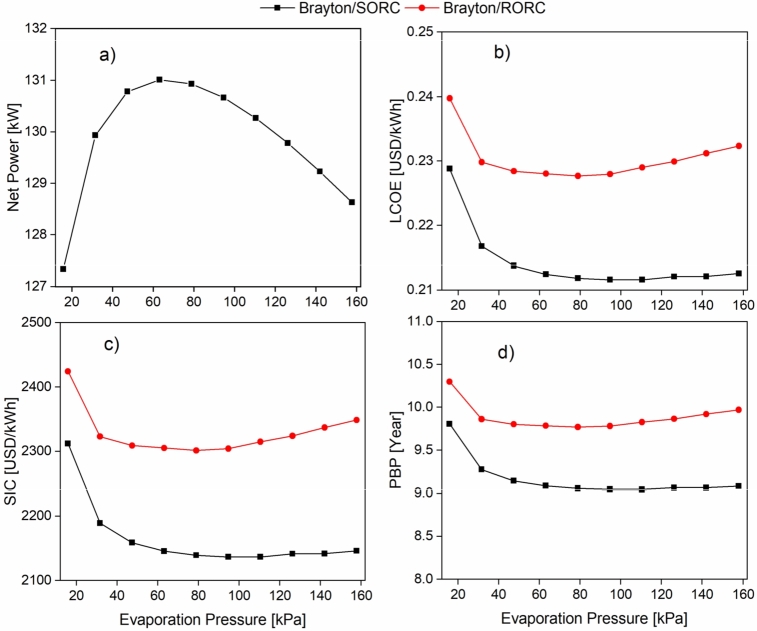
Effect of evaporation pressure on a) Wnet; b) LCOE; c) SIC; d) PBP.

On the other hand, the effect of evaporation pressure on the ORC is shown in Fig. 11. It is evident that the net power shown in Fig. 11a tends to increase at evaporation pressures between 20-63 kPa. For evaporation pressures above 63 kPa the power production tends to decrease. This is because the evaporating pressure is limited by the source temperature, i.e., the temperature of the hot fluid at the evaporator inlet. The higher the inlet temperature, the higher the evaporating pressure allowed by the evaporator due to the thermal stability of toluene. In this case, the source temperature is close to 150 °C, which admits evaporation pressures between 60 to 120 kPa. In other words, the source temperature limits the range in which the evaporation process can occur for a particular fluid, which perfectly agrees with the results presented by Meriño et al. [64]. Therefore, evaporation pressures above 160 kPa significantly decrease the net power of the system and, consequently, the thermal efficiency of the overall system.

The behavior found in Fig. 11a is reflected in the performance of the economic indicators. For example, in Fig. 11b, it is observed that as the net power increases up to an evaporation pressure of 63 kPa, the levelized cost of energy decreases. However, as the evaporation pressure increases above 63 kPa, the levelized cost increases slightly due to prevailing costs. This same behavior is exhibited by the specific investment cost (SIC), shown in Fig. 11c. It is evident that an increase in pressure causes a variation of SIC in the range between 2300 USD/kWh and 2400 USD/kWh for the Brayton/RORC configuration and between 2100 USD/kWh and 2300 USD/kWh for the Brayton/SORC configuration. Finally, the PBP shown in Fig. 11d ranges between 10.4 and 9.8 years for evaporation pressures greater than 40 kPa for the Brayton/RORC configuration, while for the Brayton/SORC configuration, the range is between 9.2 and 9.7 years.

### 3.2 Effect of decision variables on exergo economic indicators

This section explores the influence of the turbine inlet temperature, turbine efficiency, high-pressure Brayton cycle, and pressure evaporation on the exergo-economic indicators $f_{k}$ and $r_{k}$ in each component of the studied configurations. Table 7 shows the exergo-economic data obtained in their base conditions for the Brayton/SORC configuration.

**Table 7:** Exergy and exergo-economic indicators of the Brayton/SORC in the basic design.

Components	${\overset{˙}{E}}_{f.k}$	${\overset{˙}{E}}_{p.k}$	${\overset{˙}{E}}_{D.k}$	${\overset{˙}{C}}_{f}$	*ε*	*C*_*D*_	${\overset{˙}{Z}}_{f.k}$	*f*_*k*_	*r*_**k**_
	[kW]	[kW]	[kW]	[USD/GJ]	[%]	[USD/s]	[USD/s]	[%]	
Compressor	56.26	53.47	2.792	112.82	95.04	6.32 ⋅ 10^−4^	3.27 ⋅ 10^−4^	34.08	0.04
Turbine 1	65.54	63.37	2.167	86.01	96.69	1.91 ⋅ 10^−4^	2.06 ⋅ 10^−3^	91.52	0.31
Turbine 2	131.88	127.26	4.620	56.74	96.50	1.83 ⋅ 10^−4^	2.64 ⋅ 10^−3^	93.51	0.42
HTR	299.91	276.67	23.237	56.74	92.25	6.67 ⋅ 10^−4^	3.27 ⋅ 10^−4^	32.88	0.09
Heaters	1408	1401	7.37	184.29	99.48	1.46 ⋅ 10^−3^	1.41 ⋅ 10^−3^	49.28	0.26
ITC1	17.11	14.16	2.95	2478.51	82.74	5.68 ⋅ 10^−2^	7.09 ⋅ 10^−4^	1.23	0.57
Pump 1	0.06	0.01	0.05	331.59	19.49	3.92 ⋅ 10^−5^	8.97 ⋅ 10^−5^	69.52	17.28
Turbine 3	8.23	7.18	1.05	258.98	87.24	1.24 ⋅ 10^−3^	1.08 ⋅ 10^−3^	40.19	0.28
Pump 2	0.01	0.01	0.05	331.59	85.72	1.94 ⋅ 10^−5^	7.87 ⋅ 10^−5^	80.57	1.49
Evaporator	14.17	11.13	3.33	211.47	78.59	2.01 ⋅ 10^−3^	4.49 ⋅ 10^−4^	18.29	0.21
Condenser	2.97	0.06	2.43	604.80	18.20	6.88 ⋅ 10^−3^	3.82 ⋅ 10^−4^	5.42	1.25

Table 8 shows the exergo-economic values in its base conditions for the Brayton/RORC configuration. The results are similar to those obtained for the Brayton/RORC configuration. It is observed that the pump is the equipment with the highest *rk* value among the components. Therefore, the efforts should be oriented towards improving the performance of the equipment. The results also show that the turbines have an energy efficiency above 95%. While the condenser and the pump are the most inefficient equipment.

**Table 8:** Exergy and exergo-economic indicators of the Brayton/RORC in the basic design.

Components	${\overset{˙}{E}}_{f.k}$	${\overset{˙}{E}}_{p.k}$	${\overset{˙}{E}}_{D.k}$	${\overset{˙}{C}}_{f}$	*ε*	*C*_*D*_	${\overset{˙}{Z}}_{f.k}$	*f*_*k*_	*r*_**k**_
	[kW]	[kW]	[kW]	[USD/GJ]	[%]	[USD/s]	[USD/s]	[%]	
Compressor	56.26	53.47	2.792	121.37	95.04	6.80 ⋅ 10^−4^	3.27 ⋅ 10^−4^	32.45	0.04
Turbine 1	67.00	65.48	1.518	94.35	97.73	2.10 ⋅ 10^−4^	2.06 ⋅ 10^−3^	90.77	0.29
Turbine 2	134.74	131.50	3.240	62.71	97.60	2.02 ⋅ 10^−4^	2.64 ⋅ 10^−3^	92.88	0.38
HTR	297.13	274.07	23.057	62.71	92.24	7.38 ⋅ 10^−4^	3.27 ⋅ 10^−4^	30.70	0.08
Heaters	1409.00	1401.33	7.60	198.06	91.35	1.57 ⋅ 10^−3^	1.41 ⋅ 10^−3^	47.47	0.25
ITC1	15.54	12.92	2.62	2001.78	83.13	6.20 ⋅ 10^−2^	7.09 ⋅ 10^−4^	1.13	0.77
Pump 1	0.06	0.01	0.049	268.33	21.20	2.84 ⋅ 10^−5^	8.97 ⋅ 10^−5^	75.99	13.75
Turbine 3	8.19	7.14	1.046	208.00	87.23	1.22 ⋅ 10^−3^	1.08 ⋅ 10^−3^	46.99	0.29
Pump 2	0.01	0.01	0.002	268.33	85.72	1.91 ⋅ 10^−5^	7.87 ⋅ 10^−5^	80.46	1.48
Evaporator	12.93	10.53	2.401	153.77	81.44	1.25 ⋅ 10^−3^	4.49 ⋅ 10^−4^	26.39	0.14
Condenser	2.19	0.06	1.755	409.45	19.73	2.54 ⋅ 10^−3^	3.82 ⋅ 10^−4^	13.06	0.86
Regenerator	0.77	0.55	0.220	208.00	71.25	1.14 ⋅ 10^−3^	4.96 ⋅ 10^−4^	30.24	0.57

**Figure 12:**
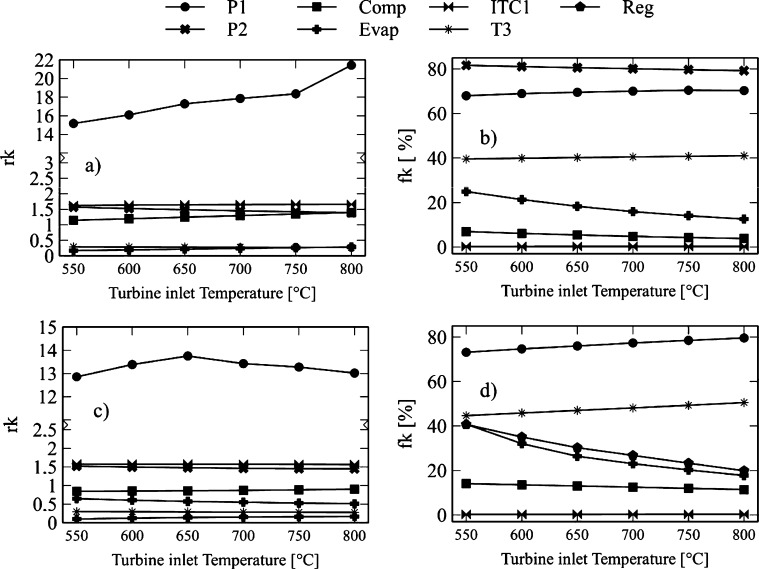
Effect of turbine inlet temperature on exergo-economic indicators: a-b) Brayton/SORC; c-d) Brayton/RORC.

**Figure 13:**
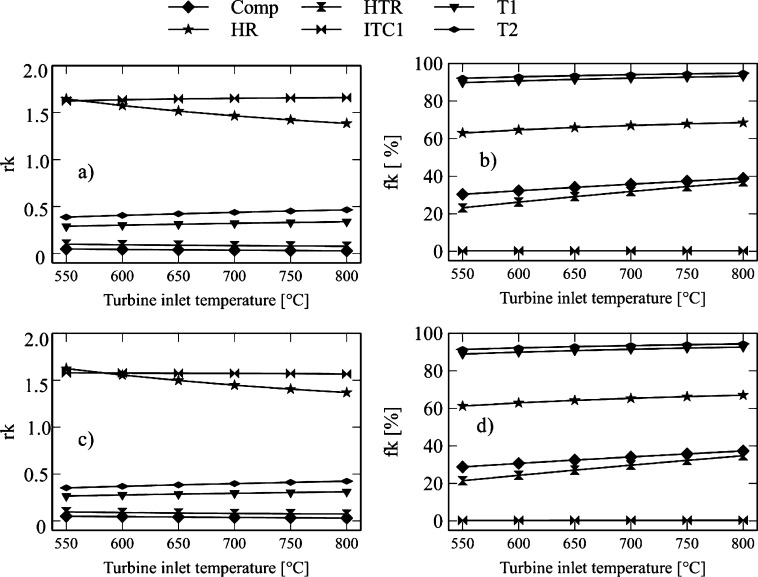
Effect of turbine inlet temperature on exergo-economic indicators: a-b) Brayton/SORC; c-d) Brayton/RORC.

Fig. 12 and Fig. 13 show the influence of turbine-1 inlet temperature on exergo-economic indicators. This variable expresses the relative increase in the average cost of exergy per unit between the input and output of the component. It also allows the identification of the sources of real costs associated with each component. Fig. 12a and Fig. 13a show the *rk* factor for the Brayton/SORC configuration. It is observed that pump-1 presents higher values concerning the other components. This fact is because the thermal oil fluid pump gives a high product cost value. The pump of the thermal coupling (P2) tends to compensate the pressure drop caused by the heat exchanger (ITC1) where the Brayton circuit fluid ($CO_{2}$) transfers heat to the thermal fluid.

Consequently, pump-1 has an increase in *rk* values in the range from 15.16 to 21.4, which corresponds to a rise of 42.28% for the Brayton/SORC configuration. When it was used the Brayton/RORC configuration (Fig. 12c and Fig. 13c), the increase was about 1.28%. Then, the HTR, Condenser, and ITC1 present values between 1.6 and 1 for the Brayton/SORC system. However, for the Brayton/RORC system, this equipment presented a small decrease. It happens because the regenerator in the ORC circuit causes a reduction in the exergy destruction due to the high use of heat. Therefore, the acquisition costs decrease because of the decrease in the thermal load required by the high-temperature fluid.

On the other hand, the exergo-economic factor is an indicator that shows the relative importance between exergy destruction and total investment costs. Low values of the exergo-economic factor of a component suggest that system savings costs can be achieved by reducing exergy destruction even if the investment capital for the component increases. Fig. 12b and Fig. 13b show the *fk* indicator for the Brayton/SORC configuration, where the heat exchanger (ITC1, HTR, and Heater) shows an increase by increasing the inlet temperature to turbine-1. These variations are less than 68% in the assessed range. It happens due to the exergy destruction associated with the heat transfer irreversibilities. There is a 50% saving potential when reducing exergy destruction rates. It is evident in the Evaporator and Condenser, where the increase of the inlet temperature of turbine-1 greatly impacts the reduction of the exergo-economic factor. It happens because the temperature increase promotes the organic fluid's thermal profile to follow the cooling water curve in the condenser. In the case of the evaporator, it is due to a small heat transfer area, which impacts the reduction of the initial investment costs. At this point, there is a high potential for financial savings when increasing the inlet temperature of turbine-1.

Besides, pumps, compressors, and turbines have higher variations concerning the heat exchanger. In the case of the Brayton/SORC system, the increase in temperature of turbine-1 causes an increase in the *fk* factor of 3.79%, 2.93%, and 3.64% for turbine-1, turbine-2, and turbine-3, respectively. It indicates that an increase in the inlet temperature of the turbine-1 causes a significant increase in the acquisition costs of these components due to the high area required. Therefore, this equipment's potential savings exist in reducing the investment costs or considering operating the turbine at operating ranges that can maintain an excellent cost-benefit ratio. Otherwise, pump-1 shows an increase of 3.34%, while pump-2 shows a reduction of 2.9%. In the case of pump-1, the growth is associated with the high work required to overcome the pressure drop, so the acquisition costs prevail. For pump-2, the exergy destruction processes predominate. Finally, the compressor experiences an increase of 28.12%, indicating that the acquisition costs associated with this component are influenced by the power required.

Finally, Fig. 12d and Fig. 13d show the *fk* values for the Brayton/RORC system. In this configuration, the *fk* values for each of the Brayton circuit components are lower than those of the Brayton/SORC system. It suggests an increase in the exergy destruction rates of the components due to the irreversibilities in these devices. However, in the ORC and thermal oil circuit, there was an increase in *fk* values, which indicates that the acquisition costs of these components increased as well. However, it is achieved at a reduction in exergy destruction rates compared to the Brayton/SORC system.

**Figure 14:**
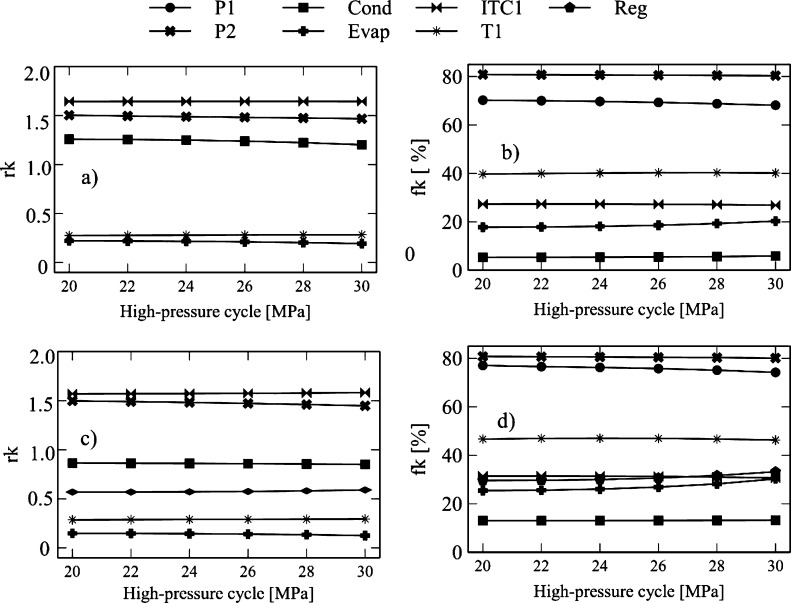
Effect of high-pressure Brayton cycle on exergo-economic indicators: a-b) Brayton/SORC; c-d) Brayton/RORC.

**Figure 15:**
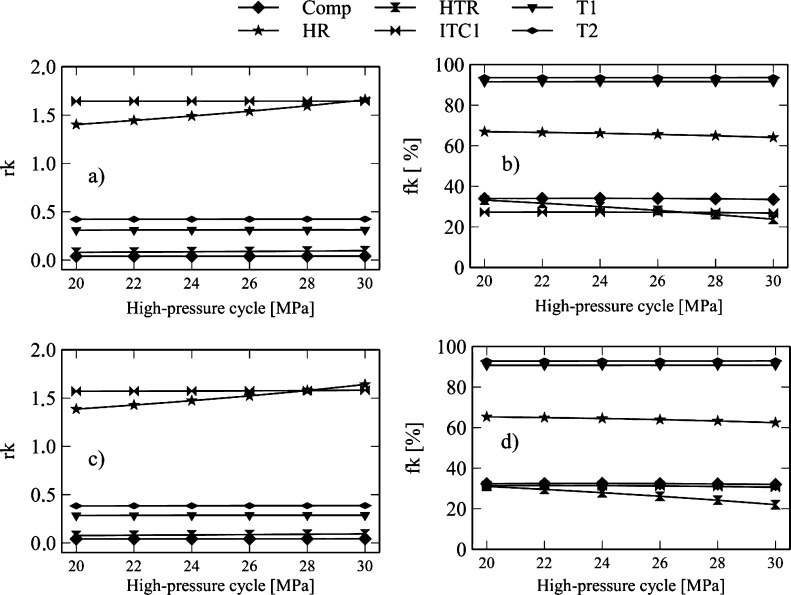
Effect of high-pressure Brayton cycle on exergo-economic indicators: a-b) Brayton/SORC; c-d) Brayton/RORC.

The effect of the high-pressure cycle on the exergo-economic indicators is shown in Figure 14, Figure 15. Fig. 14a reveals that an increase in high-pressure from 20 MPa to 30 MPa causes an increase in the *rk* factor of the compressor (4.7%), turbine-1 (0.8%), turbine-2 (0.58%), HTR (20.90%), Heater (18.26%), ITC1 (0.03%), and turbine-3 (2.92%). The increase in these values suggests that the increase in the high-pressure cycle causes an increase in the product cost. In the case of turbine-1, it happens due to the increase in the cost of power caused by pressure drops in the equipment.

Similarly, it occurs in the heat exchanger, where there is an increase in the product with respect to the input. However, there was a reduction in the *rk* values for pump-1 (11.18%), pump-2 (2.35%), evaporator (12.56%), and condenser (4.47%) in the evaluated range. It suggests a low energy quality degradation when the fluid passes through the corresponding stage.

The same behavior was found in the Brayton/RORC configuration, shown in Fig. 14c. The devices presented an increase in *rk*, e.g., for turbine-1 (0.94%), turbine-2 (0.74%), HTR (22.33%), heater (18.41%), ITC1 (0.73%), regenerator (3.78%), and turbine-3 (2.98%). The reported *rk* values were higher than those obtained in the Brayton/SORC system. It indicates that the Brayton/RORC configuration favors the increase in the product cost in a small proportion. The rest of the components of the Brayton/RORC configuration presented a high reduction in *fk*, such as pump-1 (22.66%), pump-2 (3.35%), evaporator (14.78%), and condenser (1.52%). Similar behavior was obtained in the Brayton/SORC system.

Additionally, Fig. 14b and Fig. 15b show that the increase in the high-pressure of the Brayton cycle caused an increase in the exergo-economic factor in turbine-1 (0.07%), turbine-2 (0.03%), turbine 3 (1.20%), evaporator (14.23%), and condenser (9.96%). These increments indicate that there is an increase in investment costs. The exergy destruction in the mentioned components decreases due to the high use of energy cost in the streams. A reduction in the exergo-economic factor is obtained in the compressor (1.14%), HTR (28.39%), heater (4.21%), ITC1 (1.66%), pump-1 (2.98%), and pump-2 (0.57%). In this case, the low use of the energy cost, which affects high rates of exergy destruction, predominates over investment costs.

Similar results were found for the Brayton/RORC configuration, which is shown in Fig. 14d and Fig. 15d. The increase the high-pressure made an increase in the exergo-economic factor in turbine 1 (0.09%), turbine 2 (0.05%), evaporator (19.05%), and condenser (1.22%), and regenerator (12.18%). These increments indicate that the effect of high-pressure is to increase investment costs. The destruction of exergies in these components tends to decrease due to the high use of energy costs in the streams. A reduction in the exergo-economic factor was obtained in the compressor (1.07%), HTR (28.96%), HR (4.36%), ITC1 (2.57%), pump-1 (3.71%), turbine-3 (0.92%), and pump-2 (0.83%). In this case, there is low use of energy cost, which affects high rates of exergy destruction predominating over the investment costs.

**Figure 16:**
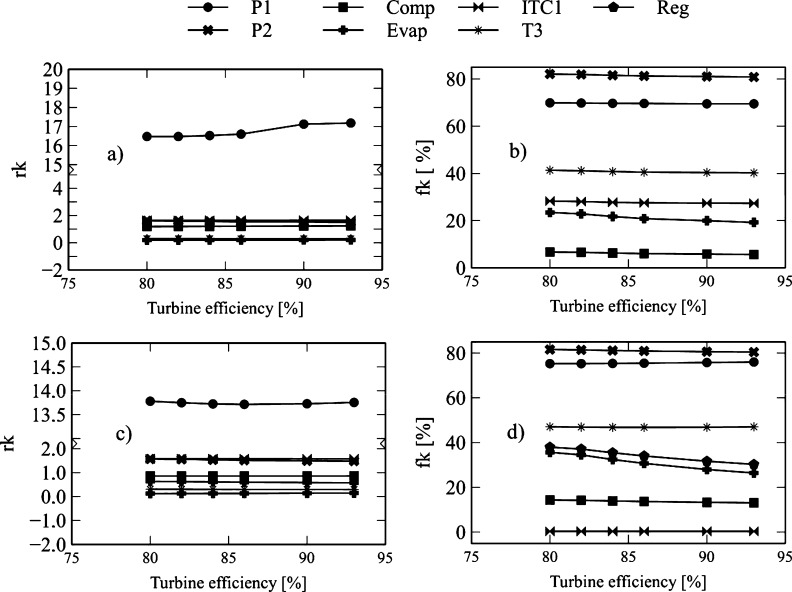
Effect of the turbine efficiency on the exergo-economic indicators: a-b) Brayton/SORC; c-d) Brayton/RORC.

**Figure 17:**
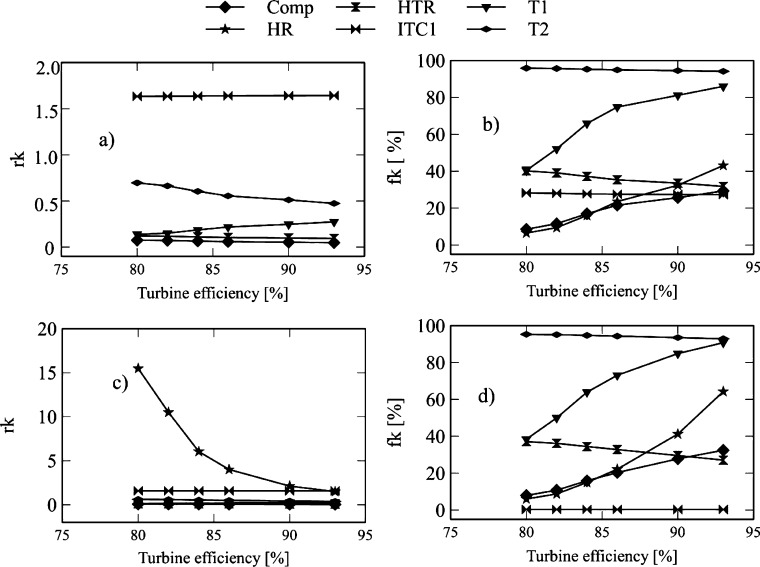
Effect of the turbine efficiency on the exergo-economic indicators: a-b) Brayton/SORC; c-d) Brayton/RORC.

Fig. 16 and Fig. 17 show the influence of the efficiency of turbine-1 of the Brayton cycle on the exergo-economic indicators. Fig. 16a and Fig. 17a reveal that the increased efficiency of turbine-1 in the Brayton/SORC configuration generates a reduction in compressor *rk* (58.7%), turbine-2 (47.1), HTR (29.3%), HR (86.2%), turbine-3 (3.9%), pump-2 (7.0%).

The reduction in these values indicates that the increased efficiency of the turbine-1 causes a degradation of the fluid power quality as it flows through the component. There is also an increase in the cost of the product of each equipment. The opposite effect was obtained in the rest of the equipment with an increase in the *rk* values. It implies an increase in the costs of flow exergies.

Fig. 16c and Fig. 17c show the *rk* values for the Brayton/RORC configuration. Similar behavior was obtained in the Brayton/SORC system. It can be inferred that the Brayton/RORC configuration tends to degrade the fluid power quality as it passes through the compressor, turbine-2, HTR, HR, ITC1, B1, turbine-3, pump-2, and regenerator.

Fig. 16b and Fig. 17b show that an increase in the efficiency of turbine-1 causes an increase in the exergo-economic factors of the compressor (70.8%), turbine-1 (53.0%), and Heater (85%). On the other hand, there is a reduction in the exergo-economic factors for turbine-2 (1.8%), HTR (26.3%), ITC1 (3.5%), pump-1 (0.6%), turbine-3 (2.7%), pump-2 (1.6%), evaporator (22.1%), and condenser (19.0%). The increase in these indicators suggests an increase in investment costs due to the increase in the efficiency of the turbine. The rate of exergy destruction does not predominate. The decrease of the exergo-economic factor implies an increase in the exergy destruction rates due to the low use of energy costs in these components. Similar results were found in the Brayton/RORC configuration shown in Fig. 16d.

**Figure 18:**
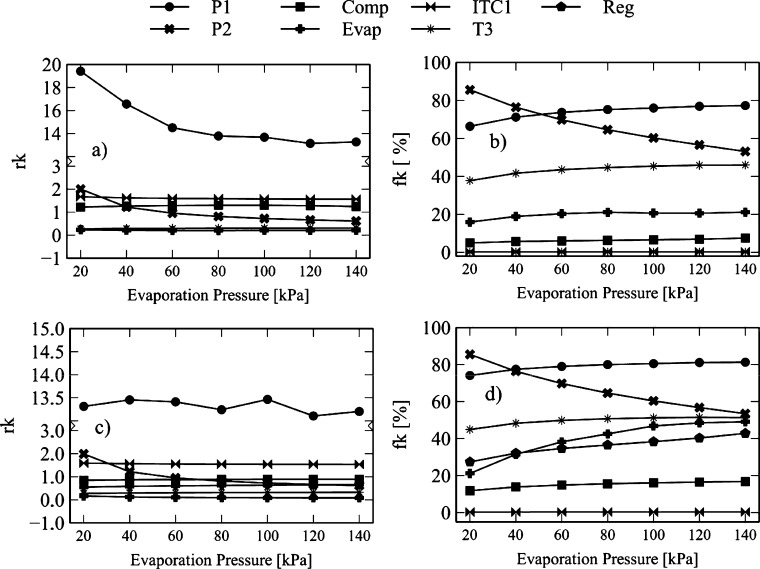
Effect of evaporation pressure on the exergo-economic indicators: a-b) Brayton/SORC; c-d) Brayton/RORC.

**Figure 19:**
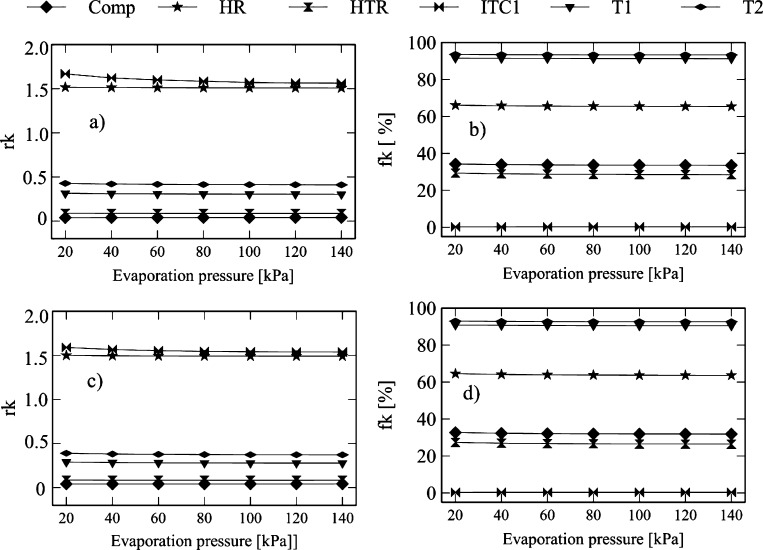
Effect of evaporation pressure on the exergo-economic indicators: a-b) Brayton/SORC; c-d) Brayton/RORC.

Fig. 18 and Fig. 19 show the influence of the evaporation pressure on exergo-economic indicators. Fig. 18a reveals that in the Brayton/SORC configuration, the increase in pressure causes an increase in the values of *rk* in the compressor (3.31%), turbine-3 (16.79%), condenser (1.99%), and a decrease in turbine-1 (3.54%), turbine-2 (3.91%), HTR (1.38%), HR (0.52%), ITC1 (6.33%), pump-1 (31.65%), pump-2 (69.43%), and evaporator (12.96%). Similar behavior happens for turbine-1, turbine-2, HTR, Heater, ITC1, pump-1, pump-2, and evaporator in the Brayton/RORC configuration using the same range of pressure, as shown in Fig. 18c and Fig. 19c. In both configurations, the results reveal that pump-1 is the component with the highest values of *rk*. This behavior happens due to the high acquisition, operation, and maintenance costs, which makes the proposed recovery system more expensive. It has to be evaluated the possibility of acquiring pumps that cause a lower increase in exergy.

Concerning the exergo-economic factor of the Brayton/SORC configuration shown in Fig. 18b and Fig. 19b, the results reveal that the increase of the pressure from 20 to 140 kPa causes a decrease in the exergo-economic factor (*fk*), e.g., in the compressor (2.02%), turbine-1 (0.34%), turbine-2 (0.28%), HTR (2.99%), HR (1.04%), and pum-2 (37.91%). A decrease in the exergo-economic factor (*fk*) was found in the ITC1 (10.72%), pump-1 (16.47%), turbine-3 (21.84%), evaporator (30.60%), and condenser (50.57%). The ORC circuit equipment is the one that has the greatest impact due to the increase in the pressure, in contrast to the ORC circuit equipment, where the variations are lower.

Similar behavior was found in the Brayton/RORC system shown in Fig. 18d. However, the obtained *fk* values in the Brayton/RORC configuration were lower than those found in Brayton/SORC configuration. It happens due to the high rates of exergy involved in the equipment. There is a potential for improvement, with particular emphasis on the pumps and heat exchangers of the ORC circuit.

### 3.3 Multi-objective optimization

The goal of the Multi-objective optimization is to maximize the net power of the system (Brayton plus ORC) by minimizing the economic costs. Three scenarios were defined to which the search algorithm was applied to obtain the Pareto points. From the Pareto frontier, the TOPSIS tool was used for the final selection of the optimal points.

**Figure 20:**
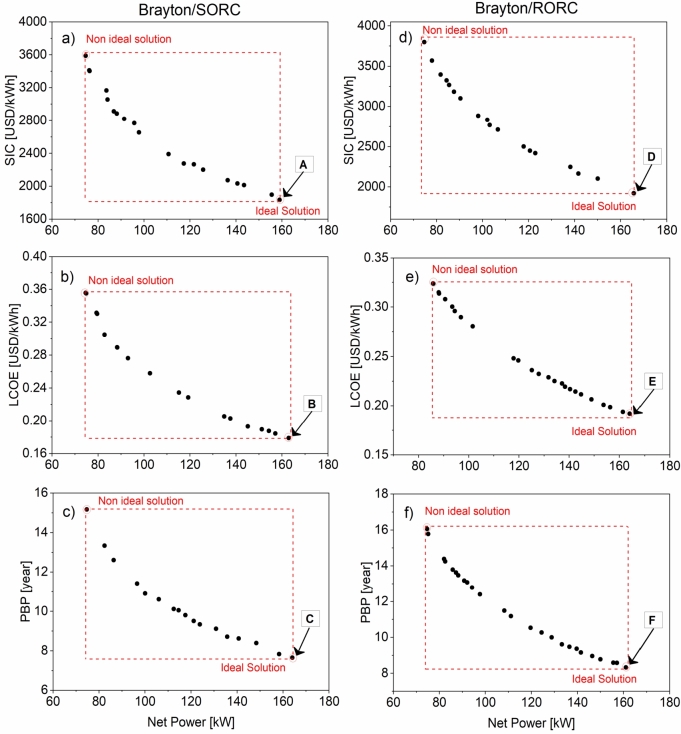
Pareto frontier points of the thermo-economic optimization for each scenario of the proposed configurations: a-c) Brayton/SORC; d-f) Brayton/RORC.

Fig. 20 shows the Pareto frontier for the three scenarios: ${\overset{˙}{W}}_{net}$/SIC, ${\overset{˙}{W}}_{net}$/LCOE, and ${\overset{˙}{W}}_{net}$/PBP, for both configurations. In each scenario, the values that maximize the net power are searched. It is observed that the Brayton/ORC configuration has higher values than the Brayton/SORC configuration in economic terms. However, considering the net power generation generated by both configurations, the results show that there is no significant difference between them. Therefore, the viability between both configurations is strongly determined by the cost, which depends on the simplicity of the system.

**Figure 21:**
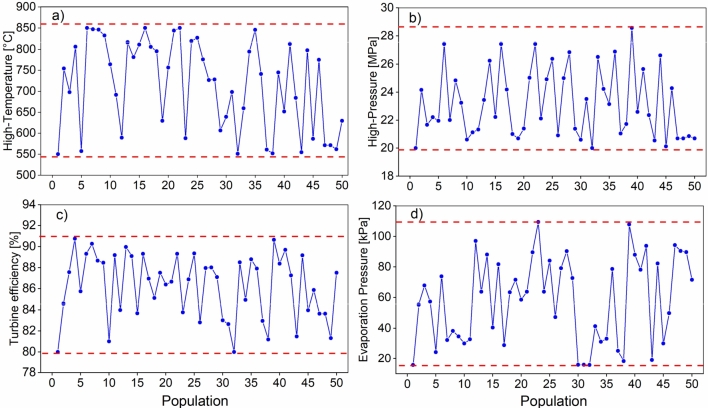
The behavior of the decision variables during optimization (${\overset{˙}{W}}_{net}$ and *SIC*) of the Brayton/SORC configuration: a) high-temperature; b) high-pressure; c) turbine efficiency; d) evaporation pressure.

The behavior of the population through the convergence process for the Brayton/SORC system is shown in Fig. 21. In this case, only the behavior of the decision variables during the optimization of the objective functions ${\overset{˙}{W}}_{net}$ and SIC is studied. In each of them, the evolution of each variable inside the search process is observed. The values of the high-temperature are inclined towards the zone between 750 °C and 800 °C, according to Fig. 21, since it favors the energetic performance of the system. The opposite is observed for the high-pressure shown in Fig. 21b in which the evolution is much more scattered, taking wider values throughout the process. However, about 50% of the points are between 24-20 MPa.

Turbine efficiency is a variable that significantly affects system performance. Fig. 21c shows that the values tend to be above 86%. Usually, an efficiency above this value is considered suitable for power systems. Finally, evaporating pressure is a critical variable limiting the ORC operability range. A significant scattering of this variable would suggest little influence on energy and economic changes. In addition, it could indicate that the algorithm is looking for values that may impact the system. In this case, the contribution that the ORC could make in terms of energy is below 15 kW at the maximum source temperature entering the evaporator.

Table 9 shows the values of the decision variables and the objective functions evaluated in their base condition. Additionally, it shows the selected points in each of the proposed scenarios for the Brayton/SORC configuration (A, B, and D) and Brayton/RORC (D, E, and F). Based on the results, it is evident that for the Brayton/SORC system, scenario B was the one that provided the best results. The values obtained for the answer variables are the lowest in contrast to the other scenarios (A and C). Comparing with the base conditions, significant reductions were obtained for the SIC (1808.50 USD/kWh-7.6%), LCOE (0.1790 USD/kWh-7.6%), and PBP (7.67 years-7.5%), with a net power increase of 10.25% equivalent to 162.93 kW.

**Table 9:** Optimization results Brayton/SORC configuration.

Parameters	Case base		Multi-Objective Optimization Brayton/SORC		
	Units	Case base	A	B	C
High-temperature	°C	750	839.20	849.83	836.75
High-pressure	MPa	25	27.94	27.44	26.86
Turbine efficiency	%	93	0.89	0.89	0.91
Evaporation pressure	kPa	78.8	111.11	89.56	87.26
Net power	kW	147.78	158.94	162.93	164.05
SIC	USD/kWh	1957.16	**1834.07**	1808.50	1854.42
LCOE	USD/kWh	0.1937	0.1815	**0.1790**	0.1836
PBP	Year	8.29	7.70	7.67	**7.65**

Therefore, concerning the Brayton/SORC system, the indicators can be significantly reduced, with turbine inlet temperature values close to 850 °C and pressures close to 27 MPa. Additionally, according to the results, an appropriate evaporation pressure value is between 90 kPa and 111 kPa. Likewise, a high turbine efficiency allows greater use of the work in the device with fewer irreversibilities.

The results of the optimization of the Brayton/RORC configuration in each of the scenarios are shown in Table 10. It is evident that scenario *D* is the one that provides the best performance in the indicators in contrast to scenarios *E* and *F*. In comparison to the base values, it was possible to reduce the economic indicators: SIC (1920.96 USD/kWh-8.7%), LCOE (1900 USD/kWh-8.7%), and PBP (8.15 years-8.6%).

**Table 10:** Optimization results Brayton/RORC configuration.

Parameters	Case base		Multi-Objective Optimization Brayton/RORC		
	Units	Case base	D	E	F
High-temperature	°C	750	827.53	836.15	838.87
High-pressure	MPa	25	21.35	29.35	28.86
Turbine efficiency	%	93	0.89	0.91	0.89
Evaporation pressure	kPa	78.8	58.71	124.88	47.61
Net power	kW	147,78	165.74	164.13	161.11
SIC	USD/kWh	2105,52	**1920.96**	1940.00	1964.34
LCOE	USD/kWh	0,2083	0.1900	**0.1919**	0.1943
PBP	Year	8.92	8.15	8.23	**8.33**

The results in Table 10 suggest that for obtaining a good performance in the Brayton/RORC configuration, it is recommended to have high turbine inlet temperatures (above 800 °C), with high pressures between 21-29 MPa, turbine efficiency between 89% and 91%, and evaporation pressure between 58-124 kPa.

However, the results show that the best performance is obtained using simple heat recovery technologies (SORC). Thus, the Brayton/SORC configuration has a greater advantage compared to the Brayton/RORC cycle due to its simplicity, which allows it to have a lower specific investment cost. In addition, the energy selling price is more economical, which will enable it to be more competitive and obtain a lower return on investment due to the use of less internal equipment.

## 4 Conclusions

A supercritical Brayton cycle with reheating coupled to a SORC and RORC system was studied from a thermodynamic (${\overset{˙}{W}}_{net}$) and economic (LCOE, PBP, SIC) point of view. The effect of four key system parameters such as turbine-1 inlet temperature, high-pressure, turbine efficiency, and evaporation pressure were considered. Also, the influence of these variables on exergo-economic indicators ($f_{k}$, and $r_{k}$) were studied. Finally, a multi-objective optimization was carried out.

It was found that the turbine-1 inlet temperature (TIT) was the variable that had the most significant effect on the economic and energy indicators of the configurations analyzed in comparison with the other. The temperature increase caused a 63.9% power increase for the Brayton/SORC and Brayton/RORC. In terms of economic indicators, the increase in temperature up to 850 °C leads to a payback period of 8.22 years for the Brayton/SORC system and 8.83 years for the Brayton/ROC system.

The other variables had a lower effect on the power generated and on the economy of the system. The high-pressure of the Brayton cycle was the variable that had the smallest effect on the exergo-economic indicators of the system components, followed by the pressure and the turbine inlet temperature. The variable with the highest impact was the turbine efficiency. The *fk* values obtained from the Brayton/RORC system were lower than those obtained with the Brayton/SORC system. The acquisition and maintenance costs of the system do not prevail in the Brayton/RORC configuration. However, there was a significant effect on the system exergy destruction associated with the increase in efficiency of the turbine-1. Similar behavior was found when studying the relative cost difference ($r_{k}$), the Brayton/RORC configuration, which presented lower values concerning the Brayton/SORC system.

Finally, from the thermo-economic optimization, it is concluded that the efficiency of these systems increases considerably with turbine inlet temperatures higher than 800 °C. In addition, the high pressure can be in the range of 25-30 MPa, and the turbine efficiency is close to 90%. It is also concluded that for supercritical Brayton systems, heat recovery technologies should be as simple as possible since they allow obtaining low levelized energy costs and good recovery periods.

## Declarations

### Author contribution statement

Guillermo Valencia Ochoa: Conceived and designed the experiments; Performed the experiments; Analyzed and interpreted the data; Wrote the paper.

Jorge Duarte Forero: Conceived and designed the experiments; Analyzed and interpreted the data; Wrote the paper.

Jhan Piero Rojas: Analyzed and interpreted the data; Contributed reagents, materials, analysis tools or data.

### Funding statement

This work was supported by 10.13039/100017432Universidad del Atlántico (Colombia) through the project named “Análisis exergo-ambiental avanzado de un sistema de recuperación de calor residual de un motor de generación mediante ORC con evaporación indirecta” through the research grant ING82-CII2019.

### Data availability statement

Data included in article/supplementary material/referenced in article.

### Declaration of interests statement

The authors declare no conflict of interest.

### Additional information

No additional information is available for this paper.
